# The co-existence of transcriptional activator and transcriptional repressor MEF2 complexes influences tumor aggressiveness

**DOI:** 10.1371/journal.pgen.1006752

**Published:** 2017-04-18

**Authors:** Eros Di Giorgio, Elisa Franforte, Sebastiano Cefalù, Sabrina Rossi, Angelo Paolo Dei Tos, Monica Brenca, Maurizio Polano, Roberta Maestro, Harikrishnareddy Paluvai, Raffaella Picco, Claudio Brancolini

**Affiliations:** 1 Department of Medical and Biological Sciences, Università degli Studi di Udine. P.le Kolbe 4—Udine Italy; 2 Department of Anatomical Pathology, Treviso General Hospital, Treviso, Italy; 3 Department of Medicine, University of Padua, Padua, Italy; 4 Experimental Oncology 1, CRO National Cancer Institute, Aviano, Italy; York University, Toronto, UNITED STATES

## Abstract

The contribution of MEF2 TFs to the tumorigenic process is still mysterious. Here we clarify that MEF2 can support both pro-oncogenic or tumor suppressive activities depending on the interaction with co-activators or co-repressors partners. Through these interactions MEF2 supervise histone modifications associated with gene activation/repression, such as H3K4 methylation and H3K27 acetylation. Critical switches for the generation of a MEF2 repressive environment are class IIa HDACs. In leiomyosarcomas (LMS), this two-faced trait of MEF2 is relevant for tumor aggressiveness. Class IIa HDACs are overexpressed in 22% of LMS, where high levels of MEF2, HDAC4 and HDAC9 inversely correlate with overall survival. The knock out of HDAC9 suppresses the transformed phenotype of LMS cells, by restoring the transcriptional proficiency of some MEF2-target loci. HDAC9 coordinates also the demethylation of H3K4me3 at the promoters of MEF2-target genes. Moreover, we show that class IIa HDACs do not bind all the regulative elements bound by MEF2. Hence, in a cell MEF2-target genes actively transcribed and strongly repressed can coexist. However, these repressed MEF2-targets are poised in terms of chromatin signature. Overall our results candidate class IIa HDACs and HDAC9 in particular, as druggable targets for a therapeutic intervention in LMS.

## Author summary

The tumorigenic process is characterized by profound alterations of the transcriptional landscape, aimed to sustain uncontrolled cell growth, resistance to apoptosis and metastasis. The contribution of MEF2, a pleiotropic family of transcription factors, to these changes is controversial, since both pro-oncogenic and tumor-suppressive activities have been reported. To clarify this paradox, we studied the role of MEF2 in an aggressive type of soft-tissue sarcomas, the leiomyosarcomas (LMS). We found that in LMS cells MEF2 become oncogenes when in complex with class IIa HDACs. We have identified different sub-classes of MEF2-target genes and observed that HDAC9 converts MEF2 into transcriptional repressors on some, but not all, MEF2-regulated loci. This conversion correlates with the acquisition by MEF2 of oncogenic properties. We have also elucidated some epigenetic re-arrangements supervised by MEF2. In summary, our studies suggest that the paradoxical actions of MEF2 in cancer can be explained by their dual role as activators/repressors of transcription and open new possibilities for therapeutic interventions.

## Introduction

MEF2 is a family of transcriptional regulators involved in the control of pleiotropic responses during development and adult life. In vertebrates four members, MEF2A/B/C/D, compose the family. MEF2 are characterized by the presence of a highly conserved N-terminal MADS/MEF2 domain involved in dimerization and DNA-binding, followed by the less conserved C-terminal transactivation region [1]. Although some actions of MEF2 are redundant, functional studies have also credited specific activities to each member of the family [2–6].

The transcriptional programs under MEF2 supervision diverge in different cell types. MEF2-targets include genes involved in various differentiation activities [7–9]. Some of these targets must be switched off, if they are not part of the ongoing differentiation program, even though a transcriptionally active MEF2 is present in the same cell. Dominant epigenetic regulations and/or the existence of multiple MEF2 transcriptional partners contribute to orchestrate the context-dependent MEF2 transcriptional landscape [3,4,8]. The four family members and their splicing variants can provide further layers of complexity to the MEF2 transcriptome [10–13]. Furthermore, MEF2 can be converted into transcriptional repressors after the binding to Cabin1, G9a or class IIa HDACs [14–16]. Among these transcriptional co-repressors, class IIa HDACs (HDAC4/5/7/9) play a pivotal role and their activity is subjected to tight cellular and environmental controls [17].

Dysfunctions in MEF2 characterize several pathological conditions, including cognitive disorders, cardiac hypertrophy and cancer [18–21]. Specifically, pro-oncogenic roles of MEF2 have been reported for certain hematological malignancies and hepatocarcinomas, which are linked to the increased expression, mutations or genetic rearrangements of these TFs [22–27]. By contrast, actions as tumor suppressors have been described in the case of soft-tissue sarcomas or in the case of mutations, mostly of MEF2B, in non-Hodgking lymphomas [11, 28–30].

The antagonistic roles of MEF2 in oncogenesis, suggested by these studies, cannot be completely explained by the context-dependent regulation of their target genes [24, 30–32]. In this scenario, the ability of MEF2 to act either as transcriptional activators or as repressors on varying the environmental and genetic backgrounds has been so far underestimated. Hence, we decided to address this point by dissecting the contribution of MEF2 to the tumorigenic process using the leiomyosarcomas (LMS) as a model. LMS are rare soft tissue sarcomas showing certain degrees of smooth muscle differentiation [33, 34]. In this manuscript we have explored the transcriptional landscape and the epigenetic modifications under the control of these TFs in relation to the tumorigenic process.

## Results

### The MEF2-HDAC axis in leiomyosarcomas

**Fig 1:**
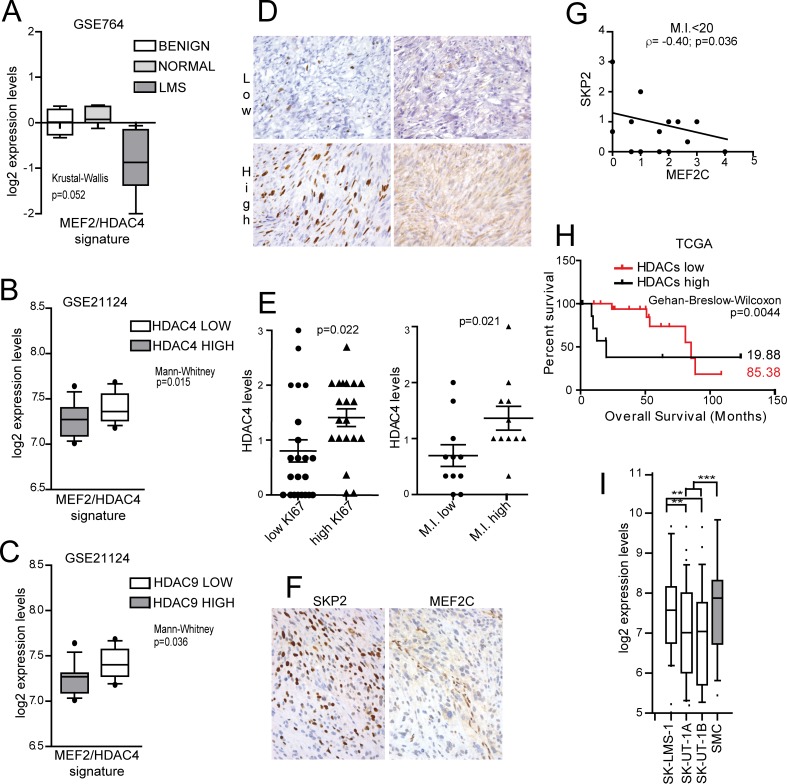
Analysis of MEF2 signature, HDAC4, SKP2 and MEF2C expression levels in leiomyosarcomas. A) Turkey box-plots illustrating the mRNA expression levels (GSE764) of MEF2-target genes in normal uterus, benign leiomyomas and malignant leiomyosarcomas. The latter are characterized by a significant (Kruskal-Wallis p<0.1) repression of MEF2 targets. B-C) The mRNA expression levels of the MEF2 targets in malignant leiomyosarcoma (GSE21124) were clustered in two sub-groups according to the expression levels of well-known MEF2 repressors (*HDAC4*, *HDAC5*, *HDAC7*, *HDAC9*, *CABIN1*). Only in the case of HDAC4 and HDAC9 a higher expression is significantly correlated to a decrease in the expression of MEF2 targets, as depicted in the Turkey box-plots. Mann-Whitney p< 0.05. D) Top pictures representing a case of uterine LMS with low Ki67 and weak and focal HDAC4 cytoplasmic expression; bottom pictures representing a case of uterine LMS with high Ki67 and diffuse cytoplasmic expression of HDAC4. E) IHC analysis of HDAC4 protein levels in LMS samples, clustered into two groups according to Ki67 positivity (1^st^ quartile = low; 3^rd^ quartile = high) (left) and M.I. (1^st^ quartile = low; 3^rd^ quartile = high) (right). Mann-Whitney p< 0.05. n = 22. F) A case of uterine LMS with nuclear expression of SKP2 in the majority of the cells and weak and focal cytoplasmic expression of MEF2C. Nuclear expression of MEF2C is present in non-neoplastic endothelial cells. Quantitative data are presented in S2 Table. G) Correlations between SKP2 and MEF2C protein levels in LMS samples, characterized by a M.I.<20. n = 20. R^2^ = 0.3. H) Kaplan-Meier survival analysis related to the expression levels of class IIa HDACs in TCGA LMS samples. From all cases (n = 106), the ones characterized by high levels of MEF2s (above the third quartile, n = 26) were analysed and clustered into two groups according to class IIa HDAC expression levels (high levels = above the third quartile, n = 8); Wilcoxon p<0.05. I) Turkey box-plots illustrating the mRNA expression levels (GSE39262) of MEF2 target genes in LMS cell lines (filled in white) compared to the normal smooth muscle cells (filled in gray). Anova p<0.05, Turkey p<0.05. * p < 0.05, ** p < 0.01, *** p < 0.001

We have previously observed that among STS, LMS evidenced the highest repression of a MEF2 signature, identified in HDAC4-transformed mouse fibroblasts and described in S1 Table [28]. Hence, we used the LMS as a model to better explore the role of MEF2 on tumor aggressiveness. Fig 1A confirms that the MEF2-signature was significantly repressed in uterine LMS compared to benign leiomyomas and normal tissues. This repression could be mediated by the activation of the PI3K/AKT/SKP2 pathway, which triggers the degradation of MEF2 proteins [35]. Alternatively, it might depend on the engagement of MEF2 transcriptional repressors. In LMS, among the different MEF2-repressors, only HDAC4 and HDAC9 mRNA levels negatively correlate with the expression of MEF2-targets (Fig 1B and 1C). We validated these data by IHC analysis, scoring HDAC4, MEF2C and SKP2 levels in a Tissue-MicroArray (TMA) of 57 LMS. HDAC4 levels were increased in tumors featuring higher proliferative activity (Ki67 positivity and high mitotic index M.I.) (Fig 1D and 1E and S2 Table). Moreover, a negative correlation between SKP2 and MEF2C was significant only in samples characterized by low (<20) M.I. (Fig 1F and 1G and S2 Table). This observation suggests that SKP2-dependent degradation of MEF2C occurs preferentially in low proliferating tumors. Interestingly, in LMS showing the highest expression of MEF2, the Kaplan-Meier analysis indicates that high levels of class IIa HDACs are associated with reduced patients’ survival (Fig 1H).

To prove the role of the MEF2-HDACs axis in LMS, we used well-established LMS cell lines. As a first step, we investigated if the repression of MEF2-target genes observed in LMS could be recapitulated in a cellular model. Two LMS cell lines, SK-LMS-1 and SK-UT-1, originally isolated from tumors with different grading (G2 and G3 respectively) [36], evidenced a robust decrease of MEF2 transcriptional activities, when compared to normal smooth muscle cells (SMC) (Fig 1I). Therefore, they could be used for our purpose.

### Differential regulation of MEF2 proteins in leiomyosarcoma cells

**Fig 2:**
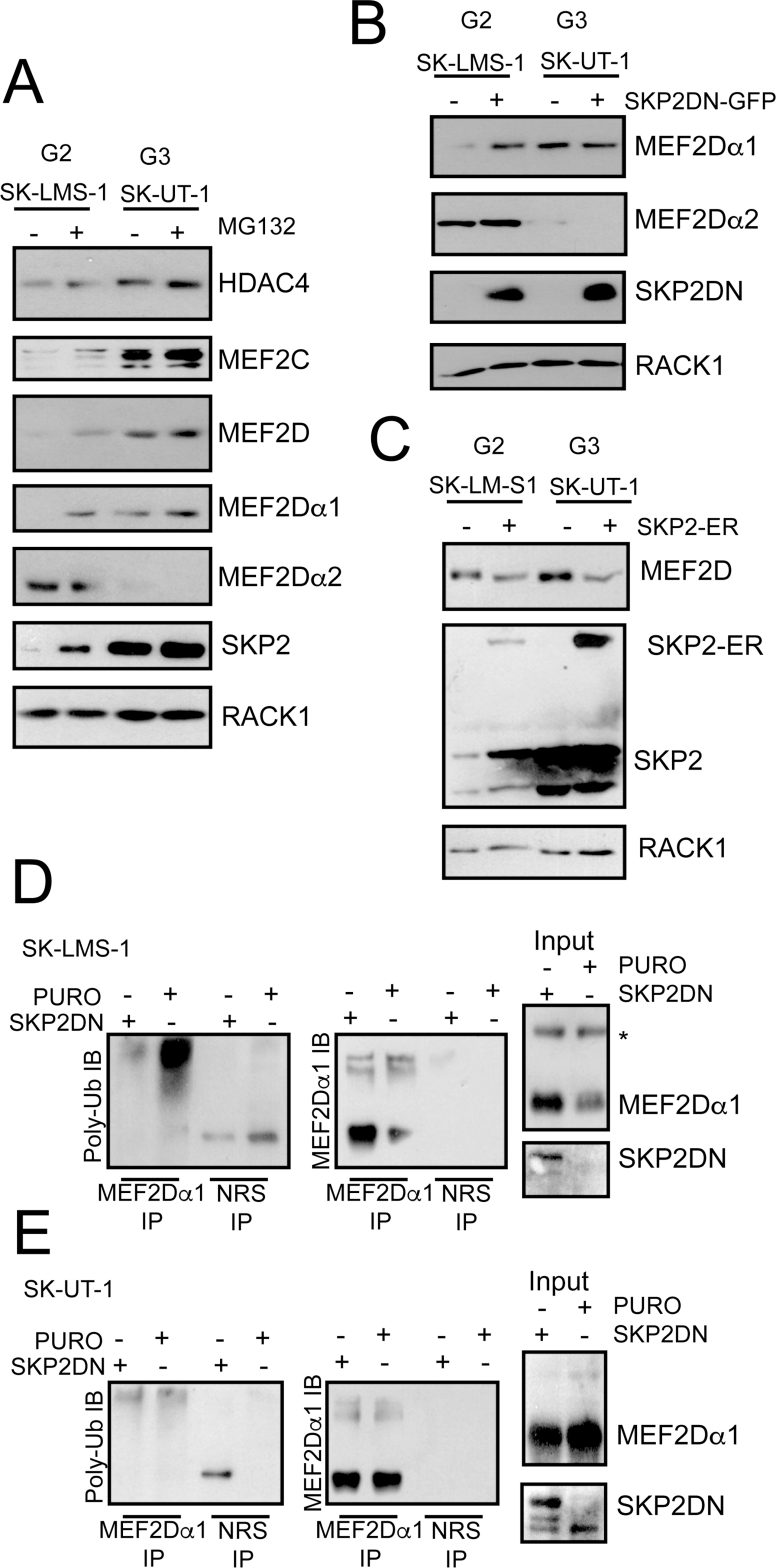
Defect in UPS-mediated MEF2-degradation in the most aggressive LMS cell line. A) Immunoblot analysis of HDAC4, SKP2 and MEF2 family members in LMS cells. Cells were treated for 8 hours with 2.5μM of the UPS inhibitor MG132. RACK1 was used as loading control. B) Immunoblot analysis of MEF2D isoforms in LMS cells engineered to express the dominant negative version of SKP2 (DN). RACK1 was used as loading control. C) Immunoblot analysis of MEF2D in LMS cells engineered to express an inducible version of SKP2 fused to ER as indicated. SKP2 was induced for 30 hours with 0.5μM 4-OHT. RACK1 was used as loading control and the nuclear relocalization of SKP2 after 4-OHT treatment was scored by immunofluorescence. D) Cellular lysates obtained in SK-LMS-1 cells expressing the DN mutant of SKP2 were immunoprecipitated using anti-MEF2Dα1 antibody and immunoblotted with the indicated antibodies. Immunoblots with total lysates (input) are also included. E) Cellular lysates obtained in SK-UT-1 cells expressing the DN mutant of SKP2 were immunoprecipitated using anti-MEF2Dα1 isoform and immunoblotted with the indicated antibodies. Immunoblots with total lysates (input) using the indicated antibodies are also included.

SK-LMS-1 and SK-UT-1 cells were characterized for the expression of MEF2D, MEF2C and HDAC4. The levels of HDAC4 were slightly increased in SK-UT-1 cells, whereas MEF2C and MEF2D levels were dramatically augmented (Fig 2A). Importantly, proteasome inhibition increased MEF2 levels only in SK-LMS-1. The UPS-independence and the high-levels of MEF2C and MEF2D in SK-UT-1 cells can be explained by the presence of a cytoplasmic retained, splicing variant of SKP2, the E3 ligase responsible for MEF2 poly-ubiquitylation [35, 37].

We also evaluated the expression of the ubiquitously expressed MEF2Dα1 isoform and of the muscle-specific splicing variant MEF2Dα2 [10]. MEF2Dα2 was expressed only in SK-LMS-1 cells (Fig 2A), in agreement with the less aggressive G2 phenotype. MG132 treatment did not influence MEF2Dα2 levels. This result indicates that the exon-switch allows escaping from inhibitory controls, probably because the α2 isoform is defective in SKP2-binding [35]. Similarly to MG132 treatment, the introduction of a dominant negative version of SKP2 (SKP2DN) augmented MEF2Dα1, but not MEF2Dα2 levels and only in SK-LMS-1 cells (Fig 2B). By contrast, introduction of an inducible version of SKP2 (SKP2-ER) diminished MEF2D levels in both cell lines (Fig 2C). Finally, poly-ubiquitylation of MEF2Dα1 can be abrogated in SK-LMS-1 cells in the presence of SKP2DN (Fig 2D), whereas this poly-ubiquitylation was almost undetectable in SK-UT-1 cells (Fig 2E). In summary, these data demonstrate that in the two LMS cells the MEF2-HDAC axis is subjected to different regulations.

### Context dependent pro-oncogenic and tumor-suppressive roles of MEF2D

**Fig 3:**
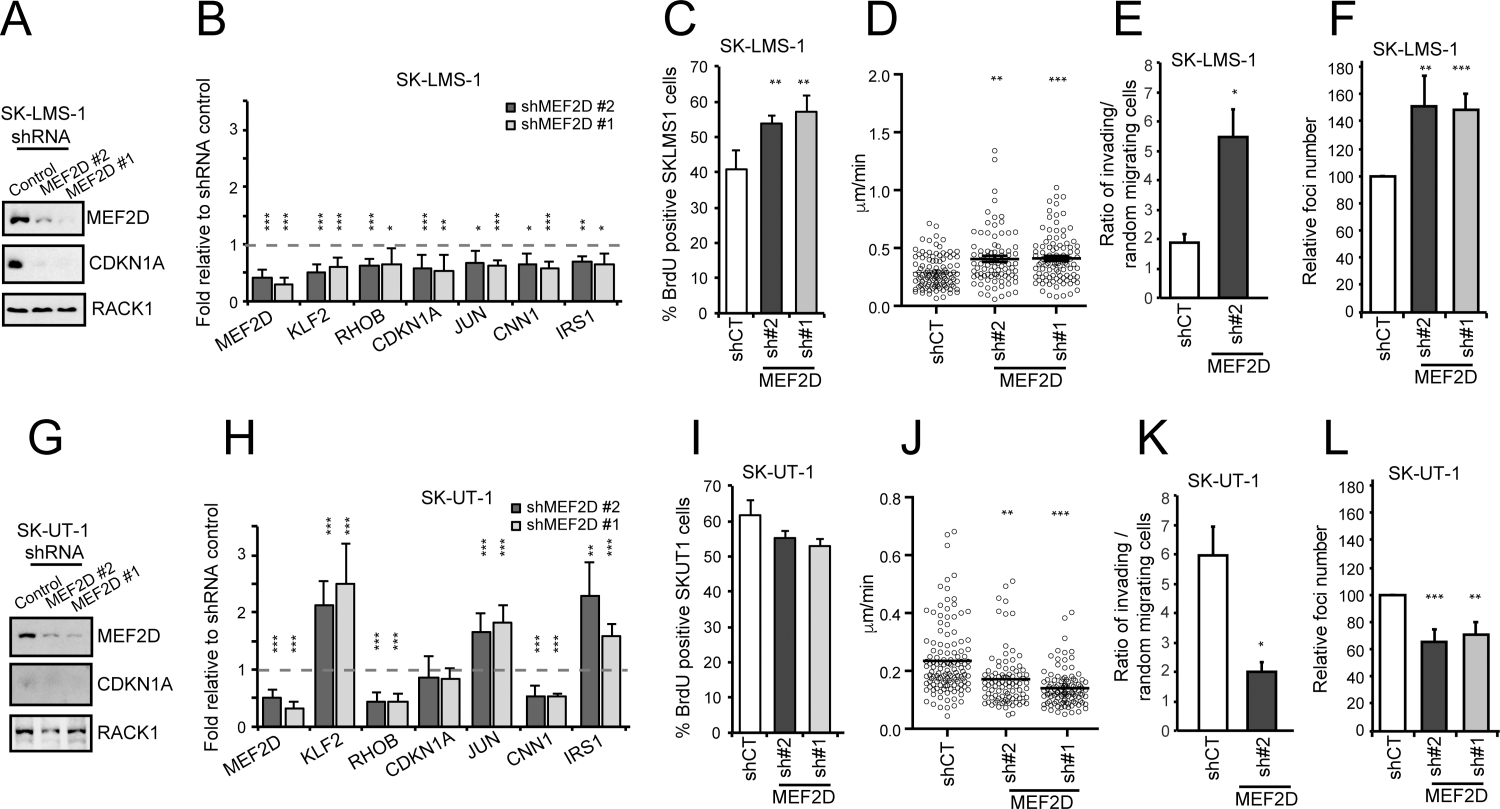
MEF2D silencing causes opposite effect in SK-LMS-1 and SK-UT-1 cells. A) MEF2D expression was silenced by lentiviral infection using two different shRNAs. Immunoblot analysis of MEF2D and CDKN1A levels in SK-LMS-1 cells expressing the control shRNA or two different shRNAs against MEF2D. RACK1 was used as loading control. B) qRT-PCR analysis of mRNA expression levels of MEF2D and of some MEF2-target genes (*KLF2*, *RHOB*, *CDKN1A*, *JUN*, *CNN1*, *IRS1*) in SK-LMS-1 cells expressing the different shRNAs. mRNA levels are relative to control shRNA. Data are presented as mean ± SD; n = 4. C) Analysis of the cells synthetizing DNA as scored after BrdU staining. Mean ± SD; n = 3. D) SK-LMS-1 cells expressing the indicated shRNAs were seeded at 2x10^4^/ml in plates coated with 10μg/ml fibronectin, or BSA; after 16h they were subjected to time-lapse analysis for 6 hours. Results represent the individual migration rate and the average (bar) from at least 140 cells from three independent experiments. Mean and SEM are indicated. E) Invasion properties of the SK-LMS-1 cells expressing the shRNA2 against MEF2D or the control. Data are presented as mean ± SD; n = 4. Invasion of the Matrigel was scored after 16 hours and was expressed as ratio between cells invading the matrix in presence (oriented motility) and absence (random invasion) of the chemoattractant. Cells were evidenced with Hoechst 33342 staining. At least 5 fields for each condition were acquired and the invading cells were counted by using ImageJ. F) Growth in soft agar of SK-LMS-1 cells expressing the indicated shRNAs, foci were stained with MTT and counted. Data are presented as mean ± SD; n = 4. G) MEF2D expression was silenced by lentiviral infection using two different shRNAs. Immunoblot analysis of MEF2D and CDKN1A levels in SK-UT-1 cells expressing the control shRNA or two different shRNAs against MEF2D. RACK1 was used as loading control. H) qRT-PCR analysis of mRNA expression levels of MEF2D and of some MEF2-target genes (*KLF2*, *RHOB*, *CDKN1A*, *JUN*, *CNN1*, *IRS1*) in SK-UT-1 cells expressing the different shRNAs. mRNA levels are relative to control shRNA. Data are presented as mean ± SD; n = 4. I) Analysis of the cells synthetizing DNA as scored after BrdU staining. Mean ± SD; n = 3. J) SK-UT-1 cells expressing the indicated shRNAs were subjected to time-lapse analysis for 6 hours as in Fig 3D. Results represent the individual migration rate and the average (bar) from at least 140 cells from three independent experiments. Mean and SEM are indicated. K) Invasion properties of the SK-UT-1 cells expressing the shRNA2 against MEF2D or the control. Data are presented as mean ± SD; n = 4. Invasion of the Matrigel was scored after 16 hours and was expressed as ratio between cells invading the matrix in presence (oriented motility) and absence (random invasion) of the chemoattractant. Cells were evidenced with Hoechst 33342 staining. At least 5 fields for each condition were acquired and the invading cells were counted by using ImageJ. L) Growth in soft agar of SK-UT-1 cells expressing the indicated shRNAs, foci were stained with MTT and counted. Data are presented as mean ± SD; n = 4. * p < 0.05, ** p < 0.01, *** p < 0.001

To clarify the contribution of MEF2 to the tumorigenic process, we knocked down (KD) MEF2D expression in SK-LMS-1 using two different shRNAs (Fig 3A). MEF2D silencing was accompanied by the down-regulation of CDKN1A. mRNA quantities of a set of MEF2-target genes (*CDKN1A*, *KLF2*, *RHOB*, *CDKN1A*, *JUN*, *CNN1*, *IRS1*) were reduced in MEF2D silenced cells (Fig 3B). In SK-LMS-1 cells, MEF2D KD increases the number of cells in S phase (Fig 3C), the random cell motility (Fig 3D) and invasiveness, as scored by *in vitro* Matrigel invasion assay (Fig 3E and S1 Fig). Finally, to complete the analysis of the tumorigenic properties, we investigated the ability to grow in soft agar. SK-LMS-1 cells with KD MEF2D develop a higher number of colonies, when grown in soft agar (Fig 3F).

When MEF2D expression was down-regulated in SK-UT-1 cells (Fig 3G) the scenario was the opposite. *CDKN1A* was not affected, and other MEF2-targets showed a heterogeneous behavior (Fig 3H). *RHOB* and *CNN1* expression was down-regulated, whereas *KLF2*, *JUN* and *IRS1* were up-regulated. DNA replication was not augmented, instead a trend toward a slight reduction was observed (Fig 3I). The random cell motility, the invasiveness properties and the growth in soft agar were all impaired in SK-TU-1 cells KD for MEF2D (Fig 3J, 3K and 3L and S1 Fig).

To exclude that the opposite tumorigenic functions of MEF2D were due to the presence of the muscle-specific MEF2Dα2 splicing variant only in SK-LMS-1 cells, we specifically silenced the MEF2Dα1 isoform. We then compared the phenotype with the KD of both α1 and α2 isoforms (S2A and S2B Fig). Expression of MEF2-target genes was similarly repressed in cells silenced for both isoforms or for only the α1 (S2C Fig). In addition, the impact on the growth in soft agar was undistinguishable between cells KD for both isoforms or only for the MEF2Dα1 splicing variant (S2D Fig).

After MEF2D, MEF2A is the MEF2 paralogue more expressed in LMS. We then asked whether also MEF2A plays a similar bi-faced role. MEF2A silencing in SK-LM-1 cells photocopied the MEF2D KD in terms of MEF2-target genes expression (S3A and S3B Fig), proliferation, cell motility, invasiveness and growth in soft agar (S3C, S3D, S3E and S3F Fig). Also in SK-UT-1 cells MEF2A silencing replicated MEF2D KD. Certain MEF2-targets were up-regulated *(KLF2*, *JUN*, *IRS1)* (S3G and S3H Fig), random cell motility, invasiveness and growth in soft agar were all impaired in MEF2A silenced cells (S3J, S3K and S3L Fig). In summary these data demonstrate that MEF2 exert opposite transforming activities in the two LMS cells.

### HDAC4 binding to promoters of MEF2-target genes is increased in SK-UT-1 cells

**Fig 4:**
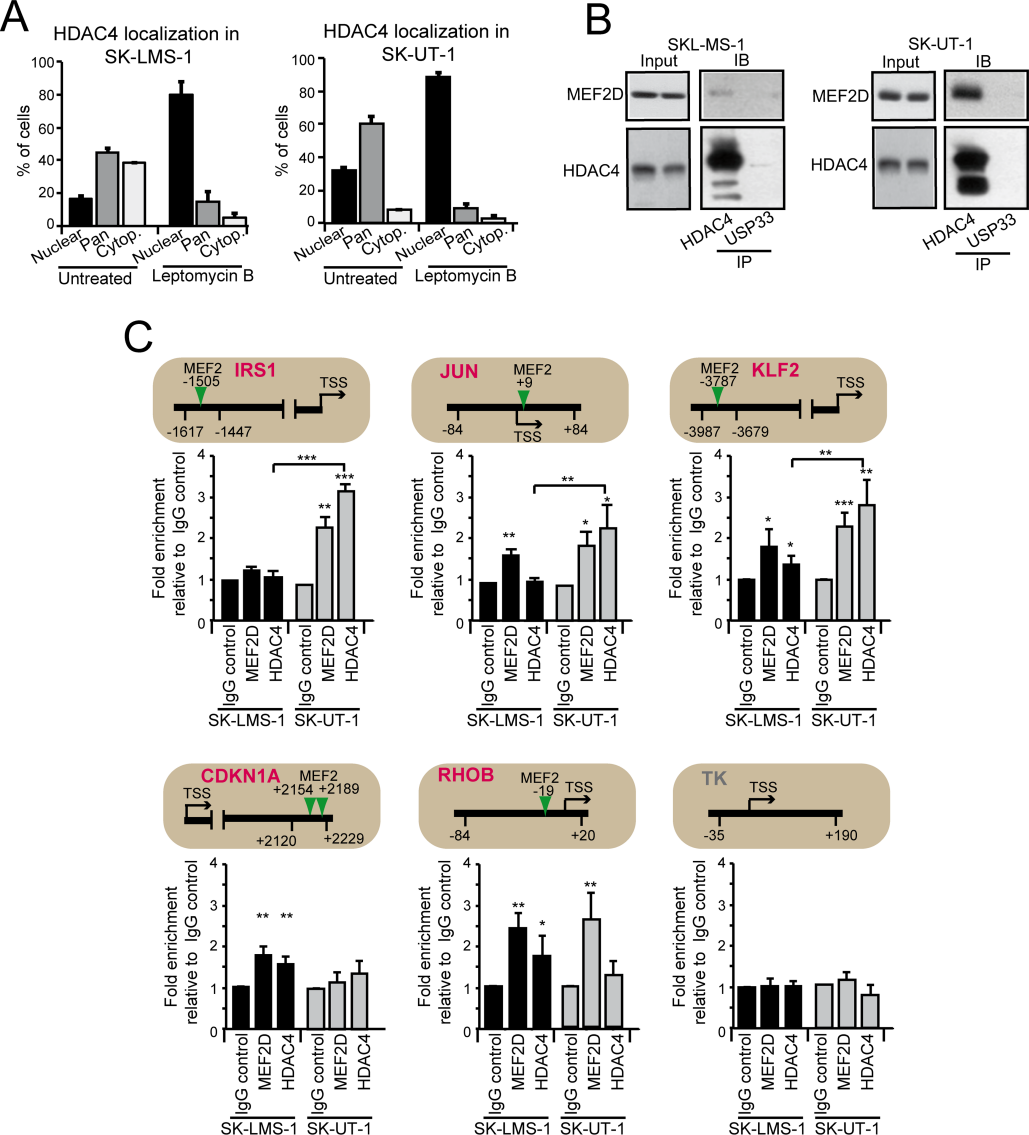
Analysis of MEF2D-HDAC4 repressive complexes in LMS cells. A) Quantitative analysis of the immunofluorescence studies. LMS cells were treated or not for 2 hours with 5ng/ml leptomycin B (LC Laboratories). After fixation of the cells, immunofluorescence analysis was performed to visualize HDAC4. Nuclei were stained with Hoechst 33342. Data are presented as mean ± SD (n = 3). B) MEF2D-HDAC4 complexes were immunoprecipitated using 1μg of anti-HDAC4, or anti-USP33, as a control, antibodies. Immunoblotting using an anti-MEF2D antibody was next used for the detection. The same amounts of cellular lysates were immunoprecipitated and the immunoblot were developed under the same circumstances. C) Chromatin was immunoprecipitated from SK-LMS-1 or SK-UT-1 cells using the anti-MEF2D and the anti-HDAC4 antibodies. Anti-FLAG antibody was used as control. *TK* promoter was used as negative control. The MEF2 binding site, the amplified region and the TSS are indicated for each tested gene, respectively with a vertical arrow, two arrowheads and a horizontal arrow. The TK promoter was used as negative control.

Our data indicate that only in SK-UT-1 cells MEF2 can exert a dominant repressive influence on certain targets. Well-known repressive partners of these TFs are the class IIa HDACs [17]. Hence, we compared the status of HDAC4 among the two cell lines. HDAC4 levels were increased in SK-UT-1 cells (Fig 2A) and also its nuclear/pan fraction (Fig 4A). In both LMS cells, HDAC4 underwent nuclear/cytoplasmic shuttling, as proved by the nuclear accumulation in response to leptomycin treatment.

Also the pool of MEF2D in complex with HDAC4 was greater in SK-UT-1 cells (Fig 4B). Finally, the binding of MEF2D and HDAC4 to the promoters of a set of well-known MEF2-target genes (*IRS1*, *JUN*, *CDKN1A*, *KLF2*, *RHOB*) between the two LSM cell lines was assessed and compared (Fig 4C). Except for *IRS1* and *CDKN1A*, the binding of MEF2D to the different promoters was similar between the two LMS cells. By contrast, the binding of HDAC4 to *JUN* and *KLF2* promoters was more pronounced in SK-UT-1 cells. Importantly, the expression of these genes was augmented in these cells after MEF2 silencing. Accordingly, binding of HDAC4 to *RHOB* promoter, whose expression was reduced in both LMS cell lines after MEF2 silencing, was undetectable in SK-UT-1 cells. Finally, ChIP-reChIP experiments confirmed the co-occupancy by MEF2D and HDAC4 of *KLF2* but not of *RHOB* promoter (S4 Fig).

### HDAC9 expression is highly induced in SK-UT-1 cells and in a relevant proportion of LMS *in vivo*

**Fig 5:**
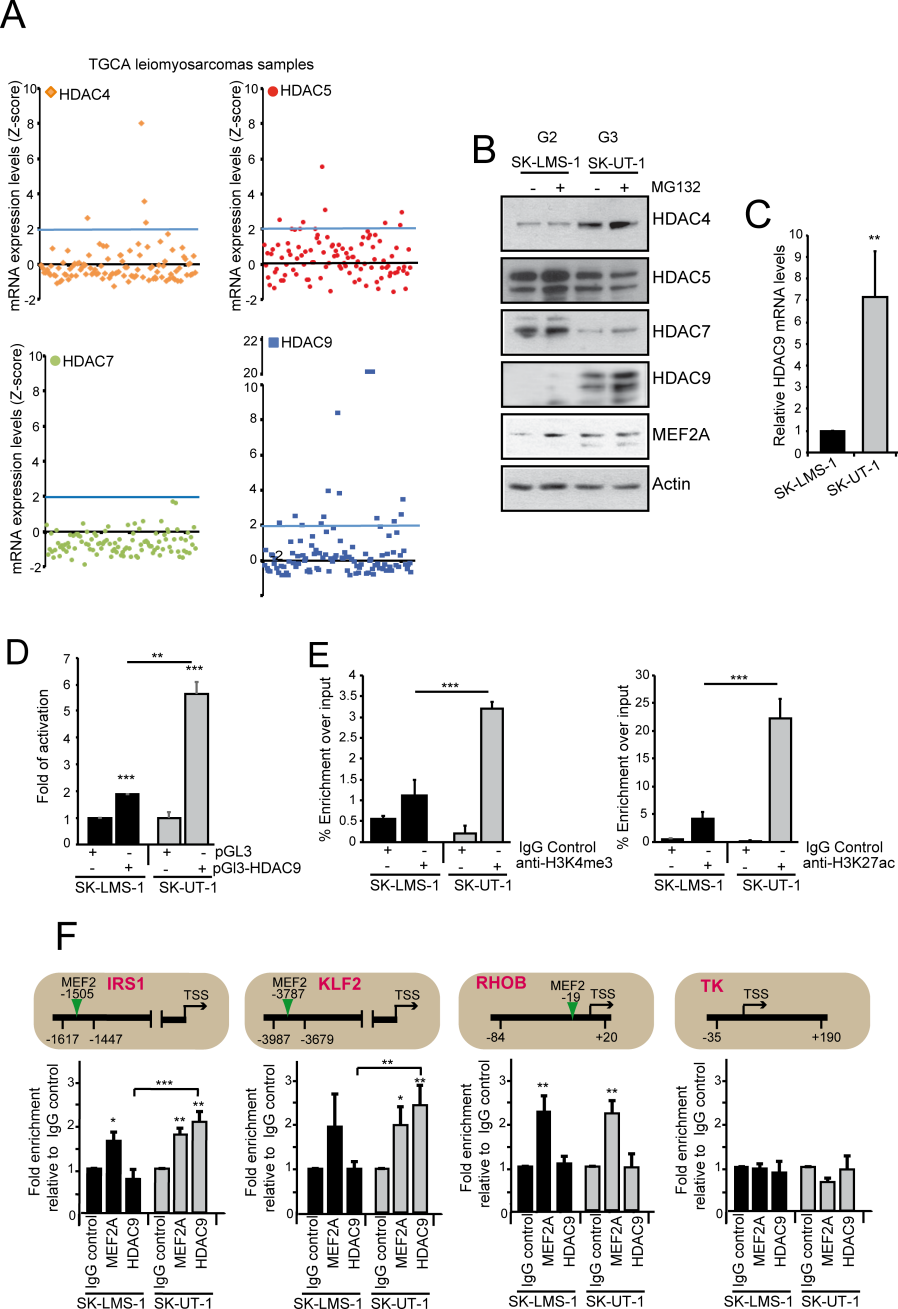
Class IIa HDACs expression in LMS. A) TCGA samples of leiomyosarcomas were analysed for the mRNA expression levels of the different class IIa HDACs. Individual tumors (n = 106) were aligned along the x axis. Data were extracted from CBio Portal (http://www.cbioportal.org/). B) Immunoblot analysis of class IIa HDACs family members and MEF2A in LMS cells. Cells were treated for 12 hours with 1μM of MG132. Actin was used as loading control. C) *HDAC9* mRNA expression levels in the two LMS cell lines. Data are presented as mean ± SD; n = 3. D) Luciferase activity after transfection in LMS cells of the empty plasmid pGL3 or the same plasmid with cloned the HDAC9 promoter isolated from SK-LMS-1 cells (bp –1160/+23). The Renilla luciferase plasmid was used as an internal control. Data are presented as mean ± SD; n = 3. E) ChIP analysis of the chromatin status in the HDAC9 promoter. Chromatin was immunoprecipitated from SK-LMS-1 or SK-UT-1 cells using the anti-H3K4me3 and anti-H3K27ac antibodies. Normal rabbit IgGs were used as control. Data are presented as mean ± SD; n = 3. F) Chromatin was immunoprecipitated from SK-LMS-1 or SK-UT-1 cells using the anti-MEF2A and the anti-HDAC9 antibodies. Normal rabbit IgGs were used as control. *TK* promoter was used as negative control. The MEF2 binding site, the amplified region and the TSS are indicated for each tested gene, respectively with a vertical arrow, two arrowheads and a horizontal arrow. * p < 0.05, ** p < 0.01, *** p < 0.001

By scrutinizing a public available database (http://www.cbioportal.org/), we noticed that also the expression of HDAC5 and HDAC9, but not of HDAC7, was augmented in certain LMS patients (Fig 5A). Collectively, approximately 22% of patients present increased expression of a class IIa HDAC member. This feature is mirrored in the SK-UT-1 cells, which are characterized by high levels of HDAC9 and by a reduction in HDAC7 expression (Fig 5B). Only in SK-UT-1 cells, proteasome inhibition increased the amount of HDAC9. Conversely, MG132 treatment stabilized MEF2A in SK-LMS-1 cells but not in SK-UT-1 cells, as above described for MEF2C and MEF2D. The high levels of HDAC9 in SK-UT-1 cells correlate with the augmented levels of the corresponding mRNA (Fig 5C). Luciferase assay using the HDAC9 promoter demonstrated that the high levels of HDAC9 in SK-UT-1 cells arise from an increased transcriptional activity (Fig 5D). ChIP on this promoter demonstrated a dramatic enrichment of an epigenetic signature (H3K27ac and H3K4me3), typical of open chromatin/active transcription only in SK-UT-1 cells (Fig 5E). Finally, ChIP experiments demonstrated a selective binding of HDAC9 to the promoters of certain MEF2-target genes (*KLF2* and *IRS1*) only in SK-UT-1 cells. By contrast, MEF2A binding to the same promoters occurs in both LMS cells (Fig 5F). In summary these data demonstrate that class IIa HDACs are overexpressed in 22% of LMS and that SK-UT-1 cells recapitulate this alteration.

### MEF2A and MEF2D-dependent transcriptional landscapes in LMS cells: definition of *classical* and *atypical* target genes

**Fig 6:**
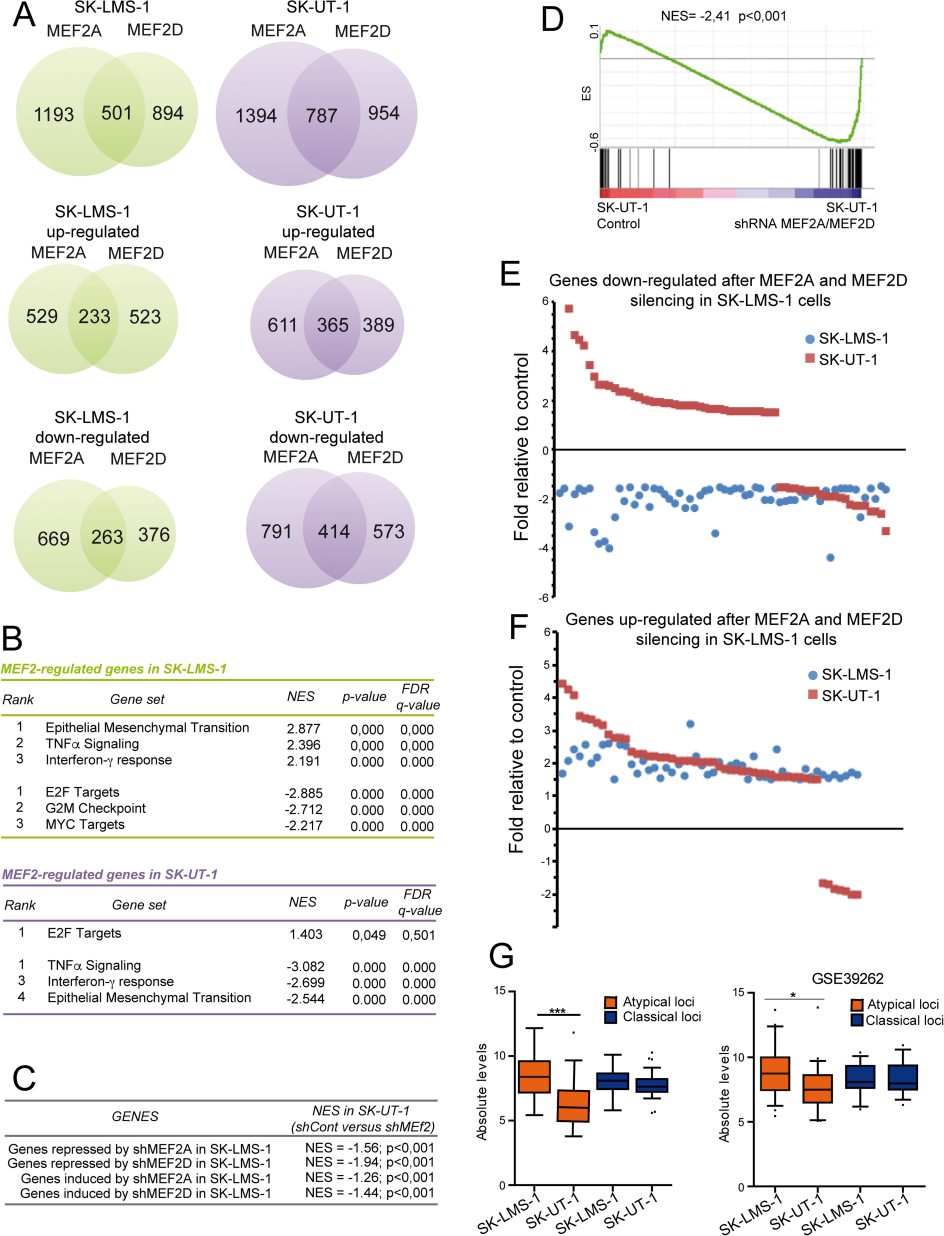
The MEF2 transcriptome. A) Venn diagrams showing the number of transcripts differing significantly in response to MEF2A and MEF2D silencing in SK-LMS-1 cells (green) or in SK-UT-1 cells (violet). Differentially expressed genes (DEGs) were selected based on fold change >1.5 and <-1.5 fold and p values <0.05. B) Gene ontology (GO) analysis was performed to interpret the principal biological processes under the regulation of MEF2 in SK-LMS-1 and SK-UT-1 cells. The NES (normalized enrichment score), the FDR (false discovery rate) and the p-value are provided. C) GSEA was performed by using SK-UT-1 DNA microarray data and genes repressed or induced by MEF2D/A KD in SK-LMS-1 cells, as indicated. Two groups were created in SK-UT-1 samples: A = shControl; B = shMEF2D/A. D) GSEA was performed by using SK-UT-1 DNA microarray data and genes repressed both by MEF2D/A KD in SK-LMS-1 and significantly modulated also in SK-UT-1 cells. Two groups were created in SK-UT-1 samples: A = shControl; B = shMEF2D/A. E) Scatter plot representing genes commonly down-regulated after MEF2A and MEF2D silencing in SK-LMS-1 cells (blue dots) and significantly modulated, after the same silencing, also in SK-UT-1 cells (red squares). F) Scatter plot representing genes commonly up-regulated after MEF2A and MEF2D silencing in SK-LMS-1 cells (blue dots) and significantly modulated, after the same silencing, also in SK-UT-1 cells (red squares). G) Turkey box-plots illustrating the mRNA expression levels of classical and atypical MEF2-target genes in SK-LMS-1 and SK-UT-1 cells in our DNA microarray and in another public available DNA microarray study (GSE39262). * p < 0.05, ** p < 0.01, *** p < 0.001

To comprehend the molecular basis responsible for the antagonistic effects of MEF2 on cancer aggressiveness, we compared the transcriptomes of the different MEF2 KD LMS cells. Several genes resulted modulated in a MEF2-dependent manner (Fig 6A). In addition to a pool of genes commonly regulated by MEF2D and MEF2A, we observed that in both cell lines some genes are under the specific regulation of one of the two paralogues (Fig 6A). For the purposes of this work we focused our attention only on genes commonly regulated by MEF2D and MEF2A. Gene-Ontology analysis revealed that in SK-LMS-1 cells, the KD of MEF2 elicits the down-modulation of genes involved in the epithelial/mesenchymal transition and in inflammation, while genes involved in proliferation and cell-cycle progression were up-regulated (Fig 6B upper part). Interestingly, both MEF2 KDs show opposite effects in SK-UT-1 cells, since genes involved in inflammation and epithelial/mesenchymal transition were instead up-regulated, while the E2F targets were repressed (Fig 6B).

Several genes down-regulated after MEF2D and MEF2A KDs in SK-LMS-1 cells were, on the opposite, up-regulated in SK-UT-1 cells after the same KDs (Fig 6C). This evidence suggests that MEF2 can preferentially behave as transcriptional activators in SK-LMS-1 cells and as transcriptional repressors in SK-UT-1 cells. To better clarify this occurrence, we focused our analysis on 85 genes, which, in SK-LMS-1 cells, were repressed after MEF2D as well as after MEF2A silencing and that were also significantly modulated in SK-UT-1 cells (|FC|>1.5, p<0.05; S3 Table).

Many (n = 58) of these 85 MEF2-target genes were up-regulated in SK-UT-1 cells after MEF2 KD (Fig 6D and 6E). We defined as “*atypical loci*” genes that were up-regulated after MEF2 silencing in SK-UT-1 cells, and as “*classical loci*” genes repressed by the KD of MEF2 in both cell lines. By contrast, the majority of genes up-regulated by MEF2 KD in SK-LMS-1 cells (n = 52) were also up-regulated (n = 45) by MEF2 KD in SK-UT-1 cells (Fig 6F). Hence, the “atypical” behavior specifically originates in SK-UT-1 cells, because of a shift towards a repressive environment under MEF2 supervision. These common MEF2-target genes, with divergent behavior, could be responsible for the antagonistic effects of MEF2 on cancer aggressiveness in the two LMS cells.

We also compared the absolute levels of expression of the classical and atypical genes between the two LMS cells. Only the atypical genes were significantly less expressed in SK-UT-1 compared to SK-LMS-1 cells, both in our microarray experiments and in another dataset (Fig 6G).

To validate the microarray studies, we performed qRT-PCR analysis on sets of classical *(ALDH2*, *ALDH6A1*, *MDX4*, *FUCA1)* and atypical *(ALPK2*, *COL1A2*, *IL8*, *SMOX*, *LEPREL1)* MEF2-targets. The expression of all classical genes was reduced in both LMS cells when MEF2A or MEF2D were silenced (S5A, S5B, S5C and S5D Fig). By contrast, only in SK-UT-1 cells the expression of the atypical MEF2-targets was up-regulated after MEF2A or MEF2D KD (S5B and S5D Fig). We also compared the binding of MEF2 to the promoters of the newly identified genes in both LMS cells. The positions of the MEF2 binding sites in the regulatory elements of these genes are shown in S4 Table. All tested MEF2-targets contain the consensus-binding site for MEF2, although at different distances from the TSS (S5E Fig). MEF2D equally binds the promoters of classical and atypical genes and this binding was dramatically reduced in MEF2D silenced cells (S5E Fig). In summary these data demonstrate that MEF2 can exert opposite transcriptional influences in different contexts.

### MEF2 differentially supervise epigenetic changes at the promoters of classical and atypical genes

**Fig 7:**
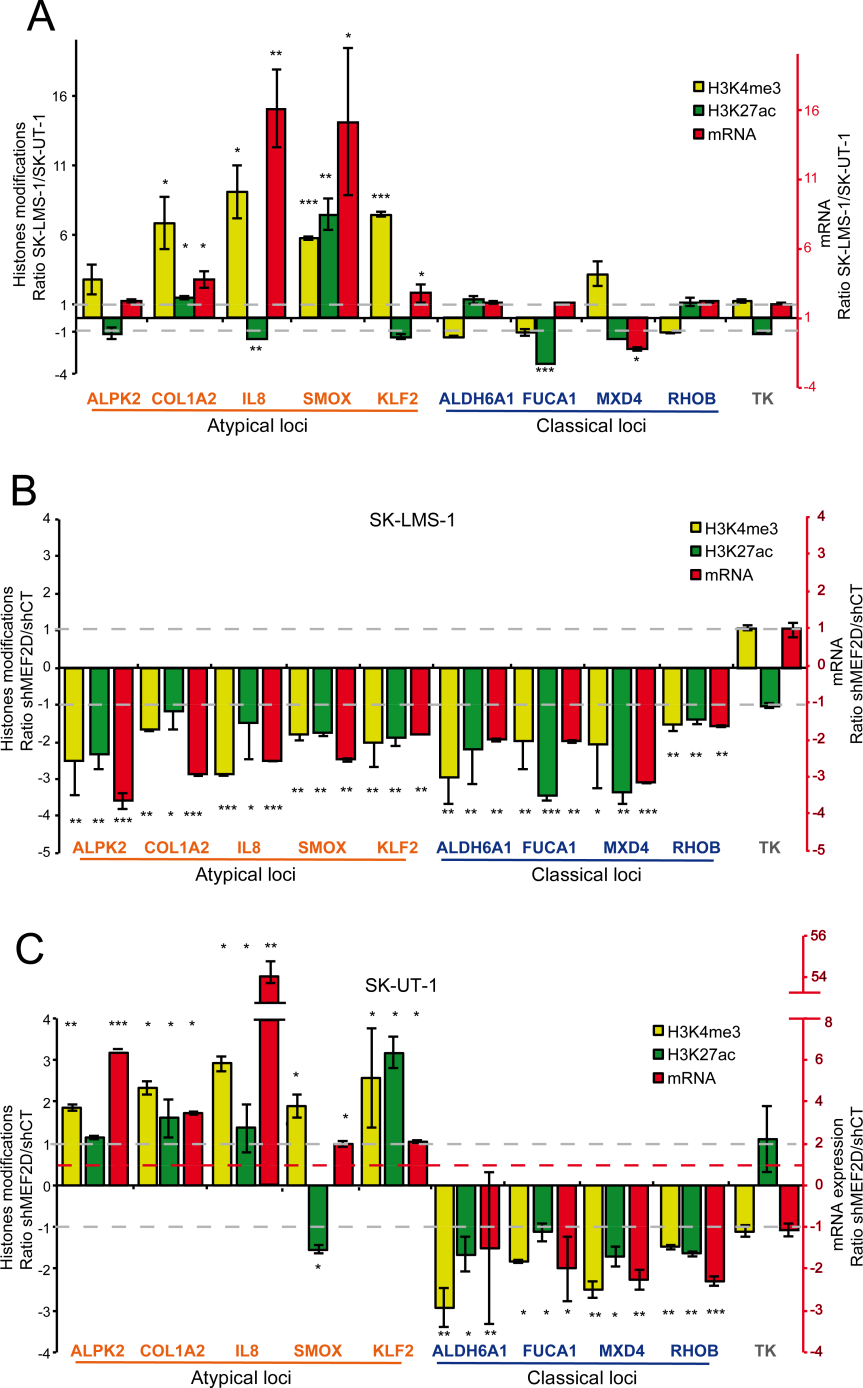
MEF2D supervised epigenetic changes on atypical and classical genes. A) Ratio between SK-LMS-1 and SK-UT-1 cells of H3K27ac, H3K4me3 and mRNA levels for a set of atypical and classical genes. *TK* was used as control. B) Ratio between SK-LMS-1 cells, WT and KD for MEF2D expression, of H3K27ac, H3K4me3 and mRNA levels for a set of atypical and classical genes. *TK* was used as control. C) Ratio between SK-UT-1 cells, WT and KD for MEF2D expression, of H3K27ac, H3K4me3 and mRNA levels for a set of atypical and classical genes. *TK* was used as control. Data are presented as mean ± SD; n ≥ 3. The binding of MEF2 was validated by ChIP (S5 Fig) and the position of binding was expressed as relative to the major TSS, according to the hg38 assembly of the human genome. * p < 0.05, ** p < 0.01, *** p < 0.001

We hypothesized that the differential transcriptional impact of MEF2 KD in the two LMS cells might reflect a specific epigenetic reprogramming. Since MEF2 can recruit HATs and HDACs onto promoter/enhancer of target genes [35, 38], levels of H3K27 acetylation were measured for a selected set of atypical genes (*ALPK2*, *COL1A2*, *IL8*, *KLF2*, *SMOX*) and of classical genes (*ALDH6A1*, *FUCA1*, *MDX4*, *RHOB)*. We also evaluated the status of H3K4me3 at the TSSs, since this modification is associated to transcriptionally active open chromatin. Data are illustrated as ratio between the two LMS cell lines and correlated to the levels of the respective mRNAs (Fig 7A). The extended data are shown in S6 and S7 Figs.

The repression of the atypical genes in SK-UT-1 cells correlated with a dramatic reduction of H3K4me3 at the respective promoters. By contrast, the levels of H3K27ac were subjected to minor fluctuations, except for *SMOX*, which promoter is much more acetylated in SK-LMS-1 cells. For all the classical genes, only minor differences were observed between the two cell lines in the case of the three investigated parameters.

After MEF2D silencing in SK-LMS-1 cells the abundance of the mRNAs, as well as H3K27ac and H3K4me3 levels, at the respective regulative regions and TSSs for all tested genes, were reduced (Fig 7B). When the contribution of MEF2D was evaluated in SK-UT-1 cells the scenario was different. mRNA levels of atypical genes were augmented, as well as H3K4me3 (Fig 7C). H3K27ac was significantly increased only for *KLF2* and *COL1A2* (modestly) and reduced for *SMOX*. In the case of the classical genes, their expression and the two histone-modifications linked to open chromatin were all reduced after MEF2D silencing, similarly to SK-UT-1 cells (Fig 7C).

Overall this analysis suggests that MEF2 can concurrently supervise H3K27 acetylation/deacetylation and H3K4 methylation/demethylation on different promoters in the same cell population.

### HDAC9 is the critical player for switching MEF2 towards a repressive influence and it is required for the transformed phenotype of LMS cells

**Fig 8:**
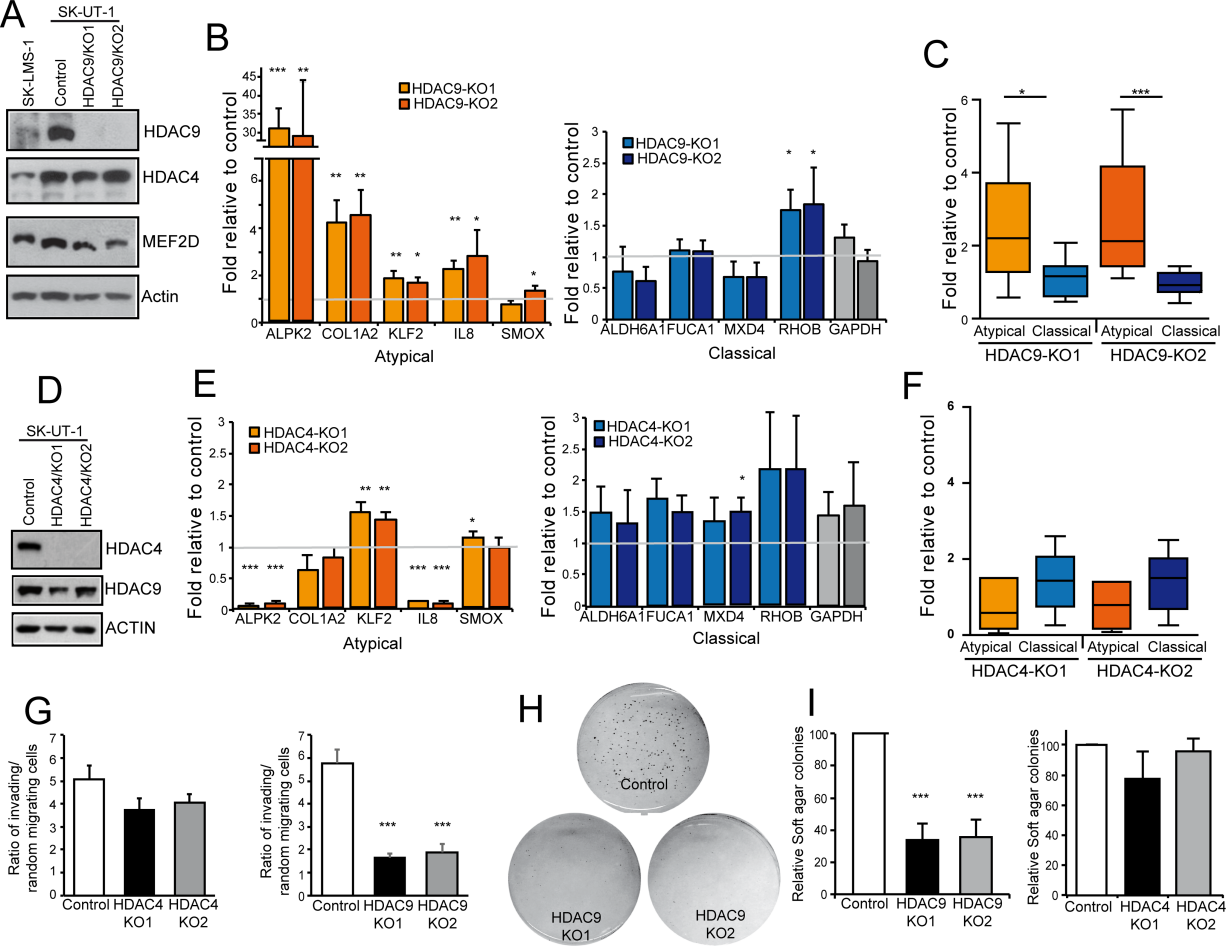
HDAC4 and HDAC9 KO in SK-UT-1 cells. A) Immunoblot analysis of HDAC4, HDAC9 and MEF2D in the indicated SK-UT-1 clones. Two different HDAC9 KO clones were selected. Actin was used as loading control. B) mRNA expression levels of the indicated atypical and classical MEF2-target genes in SK-UT-1 cells WT or KO for HDAC9. Data are presented as mean ± SD; n = 3. C) Turkey box-plots illustrating the mRNA expression levels of classical and atypical MEF2-target genes in SK-UT-1 cells WT or KO for HDAC9. Dunn's Multiple Comparison Test was applied to test the significance. D) Immunoblot analysis of HDAC4 and HDAC9 in the indicated SK-UT-1 clones. Two different HDAC4 KO clones generated by different sgRNAs were selected. Actin was used as loading control. E) mRNA expression levels of the indicated atypical and classical MEF2-target genes in SK-UT-1 cells WT or KO for HDAC4. Data are presented as mean ± SD; n = 3. F) Turkey box-plots illustrating the mRNA expression levels of classical and atypical MEF2-target genes in SK-UT-1 cells WT or KO for HDAC4. Dunn's Multiple Comparison Test was applied to test the significance. G) Invasion properties of the SK-UT-1 cells WT, KO for HDAC4 or KO for HDAC9, as indicated. Data are presented as mean ± SD; n = 4. H) Example of growth in soft agar of SK-UT-1 cells WT or KO for HDAC9. Foci were stained with MTT. I) Quantitative results of colony formation assay for SK-UT-1 cells WT, KO for HDAC4 or KO for HDAC9, as indicated. Data are presented as mean ± SD; n ≥ 3. * p < 0.05, ** p < 0.01, *** p < 0.001

The dominant repressive influence exerted by MEF2 on some promoters in SK-UT-1 cells could stem from their assembly into a repressive complex. Likely candidates for this role are HDAC4 and HDAC9. To prove this hypothesis, we used the CRISPR/Cas9 technology [39] to generate SK-UT-1 cells knock-out (KO) for *HDAC4* or *HDAC9* (S8 Fig). We analyzed in parallel two different LMS clones for each KO (Fig 8A and 8D). In SK-UT-1 *HDAC9-/-* cells the expression of the atypical genes was augmented, except for *SMOX*, which was weakly up-regulated only in the clone 2. The expression of the classical genes was not influenced by the absence of HDAC9, with the exclusion of *RHOB*, which expression was slightly augmented. Fig 8C extended this analysis to 8 atypical and 6 classical genes and confirmed the specific effect of *HDAC9* deletion on the atypical genes. Moreover, as expected, no residual binding between HDAC9 and MEF2D could be observed in SK-UT-1 *HDAC9-/-* cells (S9 Fig).

Among the atypical genes, ablation of HDAC4 provoked the modest up-regulation of only *KLF2* mRNA (Fig 8E). This is an expected result considering the capability of HDAC4 to bind its promoter (Fig 4C). Surprisingly, the KO of HDAC4 caused the down-regulation of *ALPK2* and *IL8* mRNAs (Fig 8E). These data prove that class IIa HDACs exert non-redundant functions and encourages further studies to clarify this de-regulation. The expression of classical genes was largely unperturbed in HDAC4-/- cells (Fig 8E). Box plot analysis on 8 atypical and 6 classical genes confirmed the limited impact of HDAC4 on the MEF2-dependent repressive action in SK-UT-1 cells (Fig 8F).

Finally, the tumorigenic potential of SK-UT-1 cells, as assessed in terms of invasiveness (Fig 8G) or by grow in soft agar (Fig 8H and 8I), was strongly dependent on HDAC9 and largely independent from HDAC4.

### The binding of HDAC9 to the promoter of MEF2-target genes correlates with the classical or atypical behavior

**Fig 9:**
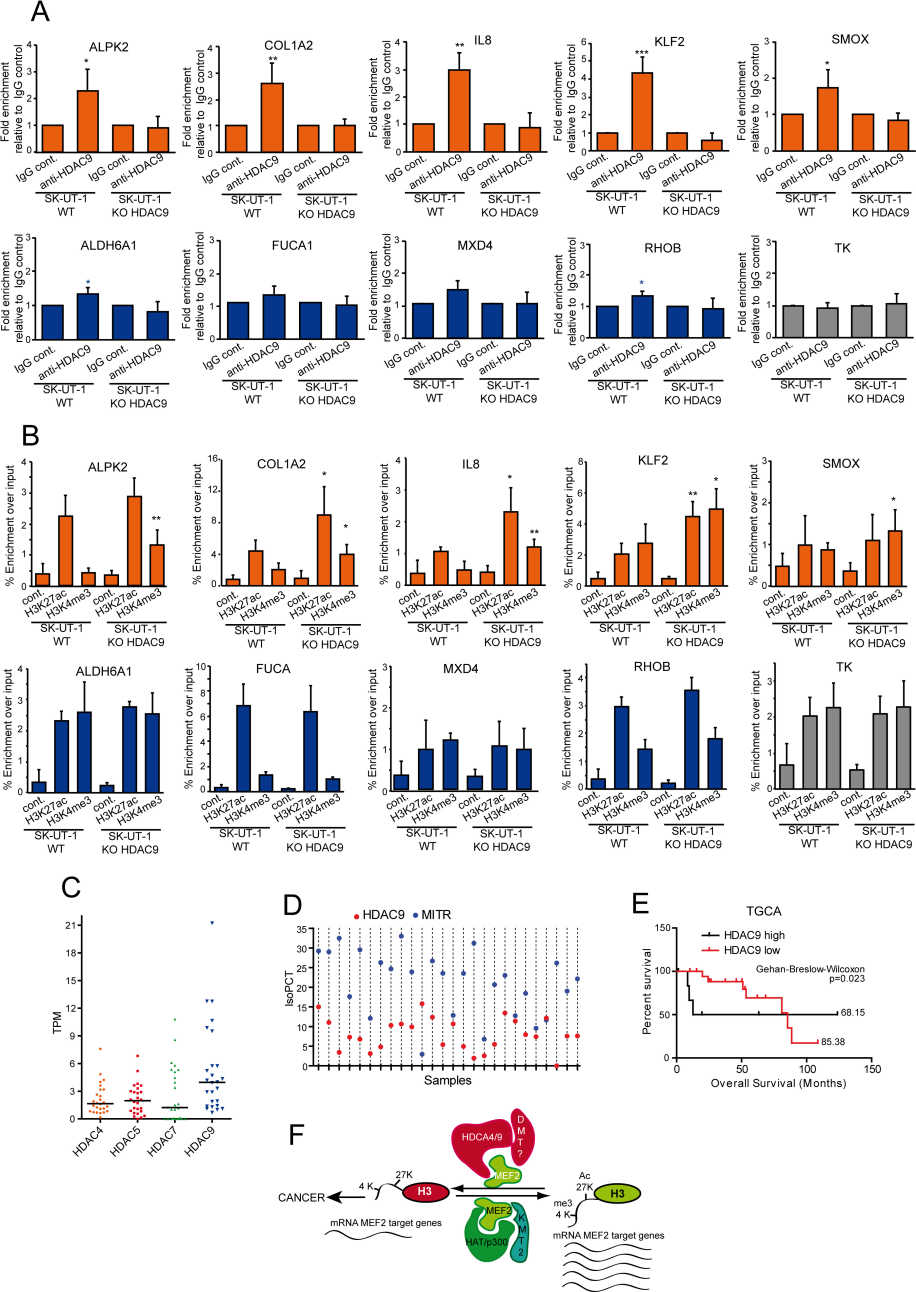
Epigenetic changes monitored by HDAC9. A) ChIP in SK-UT-1 cells WT or KO for HDAC9. Chromatin was immunoprecipitated using the anti-HDAC9 antibody. Normal rabbit IgGs were used as control. *TK* promoter was used as negative control. Atypical MEF2-targets are indicated in orange and the classical ones in blue. The genomic regions amplified by qPCR are the same as reported in S5E Fig. Data are presented as mean ± SD; n = 4. B) ChIP in SK-UT-1 cells WT or KO for HDAC9. Chromatin was immunoprecipitated using the anti-H3K27ac and the H3K4me3 antibodies as indicated. Normal rabbit IgGs were used as control. *TK* promoter was used as negative control. Atypical MEF2-targets are indicated in orange and the classical ones in blue. The genomic regions amplified by qPCR are the same as reported in S6A and S6B Fig (for H3H27ac) and in S6A and S6B Fig (for H3K4me3). Data are presented as mean ± SD; n ≥ 4. C) Scatter dot plot representing the coding mRNA TPM levels of the four class IIa HDACs in a cohort of 26 LMS samples. The horizontal lines indicate the median. Dunn's Multiple Comparison Test was applied to test the significance of HDAC9 up-regulation. D) Scatter dot plot representing the IsoPCT of the two main isoforms of HDAC9: HDAC9 WT (red) and MITR (blue). Individual tumors (n = 26) were aligned along the x axis. E) Kaplan-Meier survival analysis related to the expression levels of HDAC9, performed on the same samples and according to the same criteria as in Fig 1H. F) Summary of the shift in MEF2 transcriptional activities in relation to tumor progression in LMS. Possible co-activators and co-repressor are illustrated. The scheme describes the condition for the atypical MEF2-targets only. * p < 0.05, ** p < 0.01, *** p < 0.001.

In SK-UT-1 cells, the differential impact of MEF2 on transcription could depend on the selective recruitment of HDAC9-repressive complexes onto the promoters of atypical and classical MEF2-target genes. ChIP experiments proved that HDAC9 can be isolated, as a complex, from promoters of the atypical but not from promoters of classical genes (Fig 9A). Next, we evaluated whether HDAC9 was required to supervise H3 modifications linked to active transcription. KO of HDAC9 increased H3K4me3 content at the TSSs of the atypical genes, with again the exclusion of *SMOX*. H3K27 acetylation was increased at the promoters of *COL1A2*, *IL8* and *KLF2* (Fig 9B). When the analysis was performed on the promoters and TSSs of the classical genes, no significant changes were observed in the *HDAC9-/-* cells.

To further confirm the contribution of HDAC9 to LMS development, 26 cases of LMS were transcriptionally profiled by RNAseq and scrutinized for class IIa HDACs expression levels. Similarly to the TCGA dataset (Fig 5A), also in our LMS series HDAC9 turned out to be the most expressed class IIa HDAC (Fig 9C). Moreover, isoform quantification analyses reveled that MITR [40], the truncated HDAC9 isoform, was the more abundantly expressed (24 out of 26 samples) splicing variant in LMS (Fig 9D). Finally, the Kaplan-Meier analysis, as performed in Fig 1H but restricted to HDAC9, evidences the association of high MEF2 and HDAC9 levels with reduced patients’ survival in LMS (Fig 9E). In summary these data demonstrate that HDAC9 is an important driver of MEF2-repressive influences in SK-UT-1 cells and a key factor for the maintenance of the transformed phenotype.

## Discussion

The involvement of MEF2 in the tumorigenic process is still enigmatic [21–30, 41]. In this manuscript we provide evidences that could help to solve this controversial issue. We took advantage from the leiomyosarcoma model to unveil the two-faced of MEF2, but we are confident that these results can be confirmed also in other cancer types.

Our studies suggest that LMS can be clustered in two groups, in terms of MEF2 dysfunctions. The first group exhibited low proliferation and low levels of MEF2 proteins, possibly because of the SKP2-mediated degradation [35, 42]. The second group comprised tumors with high expression of MEF2 and of class IIa HDACs. Under these conditions MEF2 are converted into transcriptional repressors. The combination of high MEF2 and class IIa HDACs levels is detrimental for patients’ survival. Although the UPS-mediated degradation can remove MEF2 from promoters and enhancers, their conversion into repressors can provide a strongest silencing, which is translated in a worse prognosis. HDAC4, HDAC5 and HDAC9 or its splicing variant MITR are the class IIa HDACs more frequently overexpressed in LMS. A condition not limited to this tumor type, as testified by recent studies [43–47].

These observations can be recapitulated in two LMS cell lines. In SK-LMS-1 cells MEF2 are under UPS control. MEF2 ablation reduces the expression of MEF2-target genes and enhances the transformed phenotype. By contrast in SK-UT-1 cells, where HDAC9 is highly expressed, MEF2 are required for tumorigenesis and to repress the transcription of some MEF2-target genes. This conversion stems from MEF2 assembly into multi-protein repressive complexes, which erase histone marks of open chromatin configuration, such as H3K4me3 and H3K27ac, and thus repress the transcription of some MEF2-target genes (Fig 9F).

Our genes expression profile studies confirmed the complexity of the MEF2-trascriptome [2, 8, 48, 49]. Common as well as cell type and isoform specific MEF2-regulated genes exist. This multifaceted scenario could result from: i) a certain degree in DNA binding preferences of the different MEF2 [50]; ii) the presence of specific PTMs [1] and iii) the ability of forming complexes with other TFs, which hijack MEF2 away from canonical targets [4, 8].

Beside this complexity, we identified a common MEF2 signature, which can explain the opposite impact of MEF2 in the two LMS cells. Regulators of EMT, of the cell cycle and of inflammation are significantly enriched in this signature. Several genes of this signature are repressed by MEF2 in SK-UT-1 cells.

HDAC9 is the key factor for switching MEF2 into a repressor. However, HDAC9 is not recruited onto all promoters bound by MEF2. Hence, some MEF2-target genes are not repressed, even though SK-UT-1 cells express high levels of HDAC9. It is possible that these MEF2-targets govern some crucial activities of cancer cells.

The coexistence in the same cell population of TFs with both suppressive and activating activities on different loci, although surprising, was previously observed [48, 51, 52]. Different hypothesis have been formulated to explain this paradox but without a conclusive demonstration. We can exclude that the distance of the MEF2 binding site from the TSS could play a role, as previously hypothesized [48]. Since the repressive switch is cell lineage-dependent rather than gene-dependent, we could also exclude contributions of differences in the consensus of binding between promoters, as observed for p53 [52]. A fascinating hypothesis concerns contributions of the local nuclear organization, which might generate microenvironments proficient or exploitive for HDAC9 binding to MEF2.

MEF2 are required for H3K27 acetylation and H3K4 methylation. These activities can be explained by their ability to interact with Ash2L, a core subunit of KMT2 methyltransferase [53] and with the acetyl-transferase p300/CBP [54]. MEF2, once in complex with HDAC9, govern H3K27 deacetylation and H3K4 demethylation. Class IIa HDACs can bind the complex HDAC3/NuRD/SMRT, which delivers the deacetylase activity. Currently, we do not know whether a H3K4 demethylase is part of the same repressive complex with HDAC9. In principle, HDAC9 could act as a scaffold for a DMTase, as previously reported for HDAC4 and HDAC5 [55–57].

Interestingly, in SK-UT-1 cells genes repressed by MEF2-HDAC9 conserve H3K27 acetylation levels comparable to SK-LMS-1. These genes are in equilibrium between a closed and open chromatin conformation, similarly to the poised developmental regulatory genes in stem cells [58]. This condition would make possible to revert the transcriptional output of these genes by simply inhibiting the demethylase involved.

Therapeutic intervention in advanced leiomyosarcomas represents a challenge. Important obstacles are the extreme genetic heterogeneity and the relatively low incidence of these malignancies [33, 34, 59]. Here we have found that over 20% of leiomyosarcomas present increased expression of a class IIa HDAC member. *In vitro* experiments indicate that the manipulation of the MEF2-HDAC axis impinges on the transformed phenotype. In conclusion, our discoveries suggest that small molecules targeting class IIa HDACs or the interaction between the deacetylase and MEF2 could afford success for the treatment of certain LMS.

## Materials and methods

### Ethics statement

The study was conducted according to the principles expressed in the declaration of Helsinki. Written informed consent was obtained for all patients. Tissue samples were provided by the Hospital of Treviso and no additional ethics approval was needed

### Cell culture and reagents

LMS cells were grown as previously described [28]. Primary antibodies used and reagents were: anti-SKP2 8D9 (Life Technologies); anti-MEF2C [35] anti-MEF2D α1/α2 [10]; anti-GFP, anti-HDAC4 [60] and anti-HDAC5 [38]; anti-HDAC7 (sc-74563), anti-MEF2A (C-21 sc-313) and anti-RACK1 (sc-17754, Santa Cruz Biotechnology); anti-MEF2D (BD Transduction Laboratories); anti-Actin, p21 CP74 and FLAG M2 (Sigma-Aldrich); anti-ubiquitin (Covance); anti-HDAC9 (ab109446), anti-H3K27ac (ab4729) and anti-H3K27me3 (ab6002) (Abcam); anti-H3K4me3 (GTX128954, GeneTex); anti-KI67 (556003, BD Pharmingen).

### RNA extraction and quantitative qRT-PCR

Cells were lysed using TRI-REAGENT (Sigma-Aldrich). 1.0μg of total RNA was retro-transcribed by using 100 units of M-MLV Reverse transcriptase (Life Technologies). qRT-PCRs were performed using the Bio-Rad CFX96 and SYBR green (KAPA Biosystems) technology. Data were analysed by comparative threshold cycle using HPRT and β-actin as normalizer genes. All reactions were done in triplicate.

### Immunofluorescence and immunoblotting

Cells were fixed with 3% paraformaldehyde and permeabilized with 0.1% Triton X-100. Secondary antibodies were Alexa Fluor 488 anti-rabbit secondary antibodies (Molecular Probes). Actin was labeled with Phalloidin-AF546 (Molecular Probes). Cells were imaged with a Leica confocal scanner SP equipped with a 488 λ Ar laser and a 543 to 633 λ HeNe laser. Cell lysates after SDS/PAGE and immunoblotting were incubated with primary antibodies. Secondary antibodies were from Sigma-Aldrich and blots were developed with Super Signal West Dura (Pierce Waltham). For antibodies stripping, blots were incubated for 30min. at 60°C in stripping solution containing 100mM β-mercaptoethanol (Sigma-Aldrich).

### Immunoprecipitation

Co-immunoprecipitations were performed as previously described [35]. Briefly, cells were collected directly from culture dishes into RIPA buffer (50mM Tris-HCl pH8, 150mM NaCl, 0.2%SDS, 1%NP-40, 0.5%sodium deoxycholate), supplemented with protease inhibitors. Lysates were incubated for 5 h with the primary antibodies. After 1 hour of incubation with protein A beads (GE) several washes were performed. Samples were resolved by SDS–PAGE and analysed by immunoblot.

### Chromatin immunoprecipitation

ChIP experiments were performed as previously described [35]. Briefly, for each ChIP, 3x10^6^ cells were employed. DNA-protein complexes were cross-linked with 1% formaldehyde (Sigma-Aldrich) in PBS for 15 minutes at RT. After quenching and two washes in PBS, cells were collected and then lysed for 10 minutes with Lysis buffer (5 mM Pipes, 85 mM KCl, 0.5% NP40) containing protease inhibitor cocktail. Pellets were re-suspended in RIPA-100 and sonicated using Bioruptor UCD-200 (Diagenode) with pulses of 30 seconds for 15 minutes, resulting in an average size of ~500 bp for genomic DNA fragments. Samples were precleared and immunoprecipitated O/N with: 1.5μg of anti-MEF2D and anti-MEF2A, 2μg of anti-HDAC4, 4μg of anti-HDAC9, 1μg of anti-H3K27ac, 2.5 of anti-H3K4me3 and H3K27me3 antibodies or the same amount of control antibodies (FLAG M2 and USP33 serum), followed by incubation with protein A blocked with BSA and SS DNA (1μg/μl) at 4°C for 90’. Beads and inputs were treated with proteinase K at 56°C for 3h to degrade proteins and the cross-linking was reversed O/N at 68°C. Genomic DNA was finally purified with Qiagen Qiaquick PCR purification kit and eluted in 100 μl water.

### Plasmid construction, transfection, retroviral infection and silencing

pBABE-Puro plasmids expressing SKP2, SKP2DN-GFP were described previously [35]. pWZL-Hygro-SKP2 ER plasmid was obtained by subcloning with a PCR method SKP2 into pWZL-Hygro-MEF2-VP16 ER [28] and the nuclear relocalization of SKP2 after 4-OHT treatment was scored by immunofluorescence. Retroviral infections were performed as previously described [28]. pLKO-PURO plasmids expressing short hairpin RNAs (shRNAs) directed against MEF2D (15897 and 274054, referred to here as 1 and 2), MEF2Dα1 (15896, referred to here as 3) and MEF2A (432718 and 5133, referred to here as 4 and 5) were obtained from Sigma-Aldrich. pLKO-Hygro plasmid expressing the same shRNAs were obtained by oligo cloning, checked by restriction and sequencing. For lentivirus-based knock-down, HEK-293T cells were transfected with 1.8 μg of VSV-G, 5 μg of Δ8.9, and 8 μg of pLKO plasmids. After 36h at 37°C, virions were collected and opportunely diluted in fresh medium.

### Random motility measurements, invasion and soft agar assays

Random motility was assayed by time-lapse video microscopy as previously described [61]. For soft agar growth, 0.5 x10^5^ sarcoma cells were seeded in 0.3% top agar and incubated at 37°C. Foci were evidenced with MTT staining and counted by using ImageJ, as previously described [62]. For invasion assay, each well of the invasion chamber (CLS3428, Corning) was coated with 200μl of Matrigel matrix coating solution (Cultrex, Trevigen). Next, a cell suspension of 0.5x10^5^ LMS cells in 0.1%FBS-DMEM was added. As chemoattractant, 20%FBS-DMEM was added in each lower chamber. As a control 0.1%FBS-DMEM was used to evaluate random invasion.

### RNA expression array and data analysis

Total RNA was extracted using RNeasy columns (Qiagen). Aliquots of RNAs were amplified according to the specifications of the Illumina TotalPrep RNA Amplification Kit (Ambion). Hybridization on Illumina whole-genome HumanHT-12 v 4.0 chip (Illumina), scanning and background subtraction were done according to the manufacturer’s specification. Fold-change and p-values for each probe set were calculated using a moderated t-statistic in the limma package [63], with the variance estimate being adjusted by incorporating global variation measures for the complete set of probes on the array. The p-value data were then corrected for multiple hypotheses testing using the Benjamini and Hochberg.

Datasets were retrieved from GEO Dataset (GSE764, GSE21124, GSE21050, GSE39262) and analyzed as previously described [28]. For the expression levels and Kaplan–Meier analysis of TCGA sarcoma samples (265 sarcomas, 106 LMS), data were retrieved from CBio Portal [64] and expressed as z-score. Z-scores > |2| were selected as cut-off. Kaplan–Meier analysis was based on the expression levels of the four MEF2 and the four class IIa HDACs. GSEA and Gene Ontology-terms enrichment analysis were performed as described previously [28, 65].

### Tissue array construction and immunohistochemistry

Paraffin-embedded samples from leiomyosarcomas were available from 57 patients. All cases were histologically and immunohistochemically validated. Immunohistochemistry for HDAC4 (1:100), MEF2C an SKP2 was performed by an automated immunostainer (Dako Autostainer). Antigen retrieval was performed with cytrate buffer at pH 6 for HDAC4 and at pH 9 with EnVision FLEX Target Retrieval Solution (Dako) for MEF2C and SKP2. All tumors were scored for the intensity of signal (range from 0 = no expression, to 4 = strong expression). Mean of intensity and percentage of duplicate cores were used for the final analysis.

### Generation of KO SK-UT-1 cells

CRISPR/Cas9 technology was applied to obtain HDAC4 and HDAC9 clones. The KO clones were screened by PCR, immunoblot and validated by Sanger sequencing. SpCas9 and D10A mutant of spCas9 [39] were used to obtain respectively HDAC4 and HDAC9 KO clones.

### Paired-end RNA-sequencing and isoform abundancy quantification

Total RNA was extracted from FFPE sections of 26 LMS samples using the Ambion RecoverAll Total Nucleic Acid Isolation Kit (Life Technology).

RNA-sequencing libraries were prepared as previously described [66] and sequenced on a Illumina HiSeq 1000 apparatus (Illumina) to a depth of 50–80 million paired-end reads per sample. The QoRts package was used to evaluate data quality and STAR2.5.2a for reads mapping to the GRCh37.74 genome assembly. RSEM was used for quantifying gene and isoform abundances [67]. Here we provide the list of class IIa HDACs isoforms analyzed: HDAC4 (ENST00000345617, ENST00000430200, ENST00000543185), HDAC5 (ENST00000225983, ENST00000336057, ENST00000393622, ENST00000586802), HDAC7 (ENST00000080059, ENST00000380610, ENST00000427332, ENST00000552960, ENST00000354334), HDAC9 (ENST00000401921, ENST00000406451, ENST00000432645, ENST00000441542), MITR (ENST00000405010, ENST00000406072, ENST00000417496, ENST00000428307, ENST00000456174, ENST00000524023).

### Statistic

For experimental data Student t-test was employed. Mann-Whitney test was applied when normality could not be assumed. p < 0.05 was chosen as statistical limit of significance. For comparisons between samples >2 Anova test was applied, coupled to Krustal-Wallis and Dunn's Multiple Comparison Test. We mark with * p < 0.05, ** p < 0.01, *** p < 0.001. Unless otherwise indicated, all the data in the figures were represented as arithmetic mean + SD of at least three independent experiments.

## Supporting information

S1 Table Genes comprised in the MEF2 signature. (XLSX)

S2 Table Immunohistochemistry analysis. (XLSX)

S3 Table The 85 common MEF2-target genes. The list of the genes significantly regulated by MEF2A e MEF2D silencing in both SK-LMS-1 and SK-UT-1 cells. Values are indicated as mean fold change relative to the control. A prediction of the binding of MEF2 TFs on chromatin was done by scrutinizing all published BED files of ChIP-seq data: ENCFF148PLM, ENCFF001TXJ, GSE1499534, ENCFF139PSX, GSE1499535, ENCF001UPO, ENCFF001TXL, GSE73453, GSE61391, GSE43223. Pavis software was used for the annotation of the peaks [Huang W, Loganantharaj R, Schroeder B, Fargo D, Li L. PAVIS: A tool for Peak Annotation and Visualization. Bioinformatics. 2013;29: 3097–3099. doi:10.1093/bioinformatics/btt520].
(XLSX)

S4 Table Characteristics of the selected atypical and classical MEF2-target genes. (XLSX)

S1 Fig Roles of MEF2A and of MEF2D in tumor cells invasion. Fluorescence analysis of Matrigel invading SK-LMS-1 and SK-UT-1 cells expressing the indicated shRNAs and stained with Hoechst 33342. Bar = 100μM.
(TIF)

S2 Fig Silencing of MEF2D isoforms in SK-LMS-1. A) qRT-PCR analysis of the mRNAs expression levels of two alternative isoforms of MEF2D (α1 and α2) in SK-LMS-1 cells expressing the indicated isoform-specific shRNAs. mRNA levels are relative to control shRNA. Data are presented as mean ± SD; n = 3.
B) Immunoblot analysis of the MEF2D isoforms levels in SK-LMS-1 cells expressing the indicated shRNAs. Actin was used as loading control.
C) qRT-PCR analysis of the mRNAs expression levels of some MEF2-target genes (*KLF2*, *RHOB*, *IRS1*) in SK-LMS-1 cells expressing the indicated isoform specific shRNAs. mRNA levels are relative to control shRNA. Data are presented as mean ± SD; n = 3.
D) Growth in soft agar of SK-LMS-1 cells expressing the indicated shRNAs. Foci were stained with MTT and counted. Data are presented as mean ± SD; n = 4.
(TIF)

S3 Fig MEF2A silencing causes opposite effects in SK-LMS-1 and SK-UT-1 cells. A) MEF2A expression was silenced by lentiviral infection using two different shRNA (#4 and #5). Immunoblot analysis of MEF2D and CDKN1A levels in SK-LMS-1 cells expressing the control shRNA or two different shRNAs against MEF2A. Actin was used as loading control.
B) qRT-PCR analysis of the mRNA expression levels of MEF2A and of MEF2-target genes (*KLF2*, *RHOB*, *CDKN1A*, *JUN*, *CNN1*, *IRS1*) in SK-LMS-1 cells expressing the different shRNAs. mRNA levels are relative to control shRNA. Data are presented as mean ± SD; n = 4.
C) Analysis of the cells synthetizing DNA as scored after BrdU staining. Data are presented as mean ± SD; n = 3.
D) SK-LMS-1 cells expressing the indicated shRNAs were subjected to time-lapse analysis for 6 hours. Results represent the individual migration rate and the average (bar) from at least 140 cells from three independent experiments. Cell movements were quantified using MetaMorph software (Molecular Devices, Sunnyvale, CA).
E) Invasion properties of the SK-LMS-1 cells expressing the shRNA4 against MEF2A or the control. Data are presented as mean ± SD; n = 4.
F) Growth in soft agar of SK-LMS-1 cells expressing the indicated shRNAs, foci were stained with MTT and counted. Data are presented as mean ± SD; n = 4.
G) MEF2A expression was silenced by lentiviral infection using two different shRNA (#4 and #5). Immunoblot analysis of MEF2D and CDKN1A levels in SK-UT-1 cells expressing the control shRNA or two different shRNAs against MEF2A. Actin was used as loading control.
H) qRT-PCR analysis of the mRNA expression levels of MEF2A and of MEF2-target genes (*KLF2*, *RHOB*, *CDKN1A*, *JUN*, *CNN1*, *IRS1*) in SK-UT-1 cells expressing the different shRNAs. mRNA levels are relative to control shRNA. Data are presented as mean ± SD; n = 4.
I) Analysis of the cells synthetizing DNA as scored after BrdU staining. Data are presented as mean ± SD; n = 3.
J) SK-UT-1 cells expressing the indicated shRNAs were subjected to time-lapse analysis for 6 hours. Results represent the individual migration rate and the average (bar) from at least 140 cells from three independent experiments. Cell movements were quantified using MetaMorph software (Molecular Devices, Sunnyvale, CA).
K) Invasion properties of the SK-UT-1 cells expressing the shRNA4 against MEF2A or the control. Data are presented as mean ± SD; n = 4.
L) Growth in soft agar of SK-UT-1 cells expressing the indicated shRNAs, foci were stained with MTT and counted. Data are presented as mean ± SD; n = 4.
(TIF)

S4 Fig MEF2D and HDAC4 co-occupancy. 6x10^6^ cells were employed. First immunoprecipitations were conducted ON with 2μg of anti-MEF2D or anti-FLAG antibodies. Protein-DNA complexes were collected with 8μl of protein A magnetic beads (ZymoMag, Zymo research) and washed twice with RIPA and TE. Beads were incubated for 30’ at 37°C in Re-Chip elution buffer (1×TE, 2%SDS, 15mM DTT), diluted 15 times into RIPA buffer and subjected to the second immunoprecipitation using 3μg of anti-HDAC4 or USP33 IgG as control. *TK* promoter was used as negative control. Data are presented as mean fold enrichment relatively to the first input
(TIF)

S5 Fig Characterization of the new MEF2-target genes. A) qRT-PCR analysis of the mRNA expression levels of the identified atypical and classical MEF2-target genes in SK-LMS-1 cells expressing the shRNAs against MEF2D. mRNA levels are relative to control shRNA. *GAPDH* was used as control. Data are presented as mean ± SD; n = 3.
B) qRT-PCR analysis of the mRNA expression levels of the identified atypical and classical MEF2-target genes in SK-UT-1 cells expressing the shRNAs against MEF2D. mRNA levels are relative to control shRNA. *GAPDH* was used as control. Data are presented as mean ± SD; n = 3.
C) qRT-PCR analysis of the mRNA expression levels of the identified atypical and classical MEF2-target genes in SK-LMS-1 cells expressing the shRNAs against MEF2A. mRNA levels are relative to control shRNA. *GAPDH* was used as control. Data are presented as mean ± SD; n = 3.
D) qRT-PCR analysis of the mRNA expression levels of the identified atypical and classical MEF2-target genes in SK-UT-1 cells expressing the shRNAs against MEF2A. mRNA levels are relative to control shRNA. *GAPDH* was used as control. Data are presented as mean ± SD; n = 3.
E) Chromatin was immunoprecipitated from SK-LMS-1 or SK-UT-1 cells using the anti-MEF2D antibody. Anti-FLAG antibody was used as control. Cells KD for MEF2D are indicated. *TK* promoter was used as negative control. The MEF2 binding site (arrowheads), the amplified region and the TSS (arrows) are indicated for each tested gene.
Atypical MEF2-target genes are in orange whereas classical ones are in blue. Data are presented as mean ± SD; n = 3.
(TIF)

S6 Fig Role of MEF2 in controlling histone H3K27 acetylation. A) Chromatin was immunoprecipitated from SK-LMS-1 or SK-UT-1 cells WT or KD for MEF2D, using the anti-H3K27ac antibody. Normal rabbit IgGs were used as control. The MEF2 binding site (arrowheads), the amplified region and the TSS (arrows) are indicated for each tested atypical gene. Data are presented as mean ± SD; n = 3.
B) Chromatin was immunoprecipitated from SK-LMS-1 or SK-UT-1 cells WT or KD for MEF2D, using the anti-H3K27ac antibody. Normal rabbit IgGs were used as control. *TK* promoter was used as negative control. The MEF2 binding site (arrowheads), the amplified region and the TSS (arrows) are indicated for each tested classical gene. Data are presented as mean ± SD; n = 3. Atypical MEF2-target genes are in orange whereas classical ones are in blue.
(TIF)

S7 Fig Role of MEF2 in controlling histone H3K4 methylation. A) Chromatin was immunoprecipitated from SK-LMS-1 or SK-UT-1 cells WT or KD for MEF2D, using the anti-H3K4me3 antibody. Normal rabbit IgGs were used as control. The MEF2 binding site (arrowheads), the amplified region and the TSS (arrows) are indicated for each tested atypical gene. Data are presented as mean ± SD; n = 3.
B) Chromatin was immunoprecipitated from SK-LMS-1 or SK-UT-1 cells WT or KD for MEF2D, using the anti-H3K4me3 antibody. Normal rabbit IgGs were used as control. *TK* promoter was used as negative control. The MEF2 binding site (arrowheads), the amplified region and the TSS (arrows) are indicated for each tested classical gene. Data are presented as mean ± SD; n = 3. Atypical MEF2-target genes are in orange whereas classical ones are in blue.
(TIF)

S8 Fig CRISPR/Cas9 mediated KO of HDAC4 and HDAC9. A) Schematic representation of *HDAC9* genomic organization with indicated: the exons (vertical bars), the introns (junctions between the bars) and the PAM sequences utilized for the CRISPR approach.
B) Genomic sequences of the *HDAC9*-/- SK-UT-1 cells used in this study. The sequence of *HDAC9* genomic region targeted by the CRISPR/Cas9D10A is included. The PAMs and the two gRNAs are underlined. SKUT-1 HDAC9 KO clones were obtained through the delivery of the D10A mutant of SpCas9. Two sgRNAs designed on the second coding exon of HDAC9 were co-delivered to obtain two close cleavages on the genome to simulate a DSB (sgRNA1: CTGCTATCAGAAGCTGCTTC; sgRNA2: GAACTTGACACGGCAGCACC). Five clones were selected for the presence of deletions or insertion and among them the indicated two were selected for the analysis.
C) Schematic representation of *HDAC4* genomic organization with indicated: the exons (vertical bars), the introns (junctions between the bars) and the PAM sequences utilized for the CRISPR approach.
D) Genomic sequences of the *HDAC4*-/- SK-UT-1 cells used in this study. The sequence of *HDAC4* genomic region targeted by the CRISPR/Cas9 is included. The PAMs and the two gRNAs are underlined. SKUT-1 HDAC4 KO clones were obtained through the delivery of wild-type spCas9 (pLENTI-CRISPRv2). Two sgRNAs designed on the second coding exon were used (sgRNA1: GCAGGATTCAGCAGCTCCAC; sgRNA2: CGTGAACCACATGCCCAGCA). One and three KO clones were obtained respectively with sgRNA1 and 2. The two representative clones indicated here were selected for the analysis.
(TIF)

S9 Fig MEF2D-HDAC9 complex. The MEF2D-HDAC9 complexes were immunoprecipitated from the different cell lines using 1μg of anti-MEF2D, or anti-FLAG antibodies, as a control. Immunocomplexes were subjected to immunoblotting using the anti-MEF2D and HDAC9 antibodies.
(TIF)
