# Supplementary figures and images for: The co-existence of transcriptional activator and transcriptional repressor MEF2 complexes influences tumor aggressiveness
Source: PLoS Genet. 2017 Apr 18;13(4):e1006752. doi: 10.1371/journal.pgen.1006752 (PMC5413110; doi:10.1371/journal.pgen.1006752)

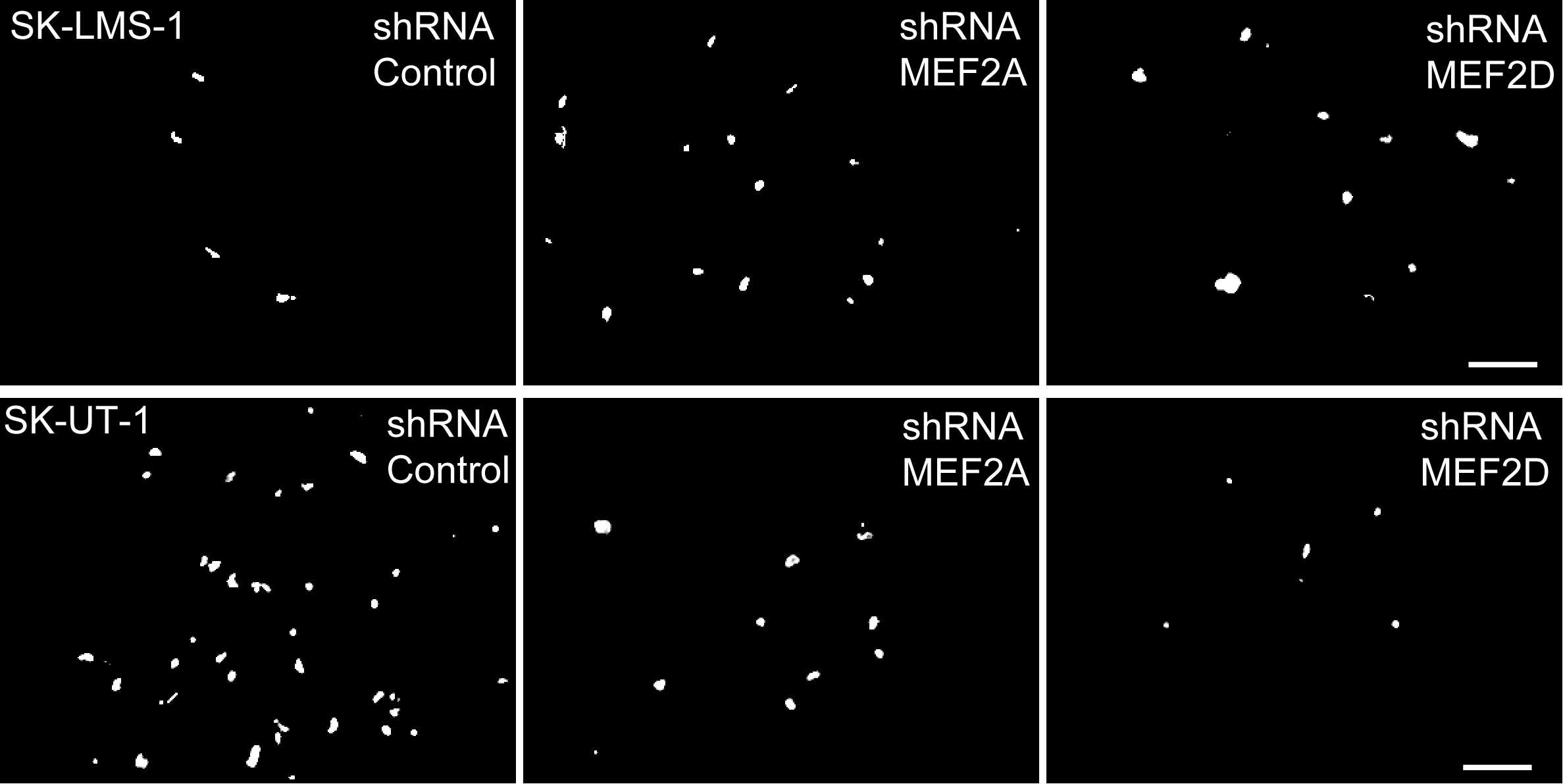

Supplement: S1 Fig — Fluorescence analysis of Matrigel invading SK-LMS-1 and SK-UT-1 cells expressing the indicated shRNAs and stained with Hoechst 33342. Bar = 100μM. (TIF) [file pgen.1006752.s005.tif]

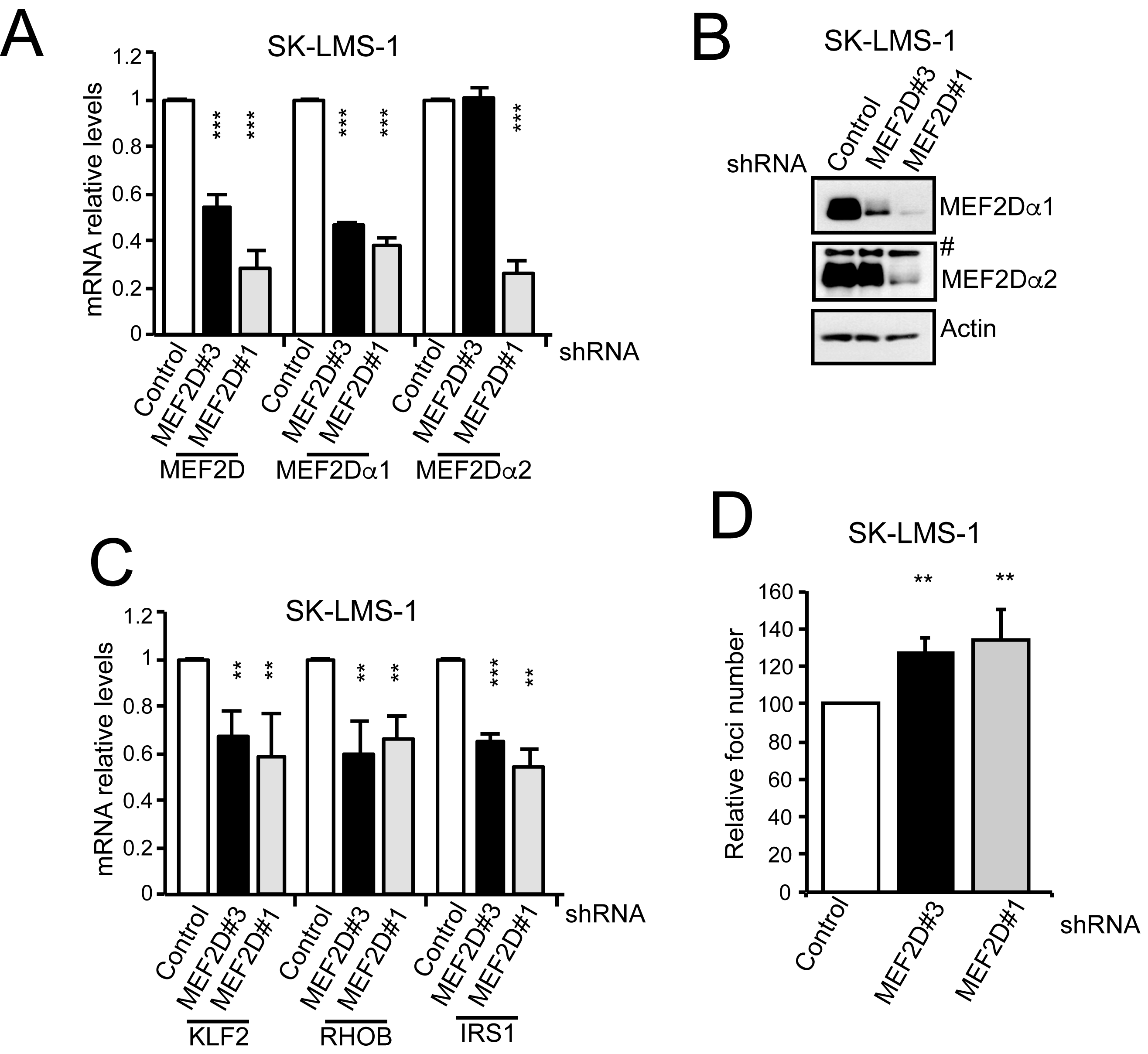

Supplement: S2 Fig — A) qRT-PCR analysis of the mRNAs expression levels of two alternative isoforms of MEF2D (α1 and α2) in SK-LMS-1 cells expressing the indicated isoform-specific shRNAs. mRNA levels are relative to control shRNA. Data are presented as mean ± SD; n = 3. B) Immunoblot analysis of the MEF2D isoforms levels in SK-LMS-1 cells expressing the indicated shRNAs. Actin was used as loading control. C) qRT-PCR analysis of the mRNAs expression levels of some MEF2-target genes (KLF2, RHOB, IRS1) in SK-LMS-1 cells expressing the indicated isoform specific shRNAs. mRNA levels are relative to control shRNA. Data are presented as mean ± SD; n = 3. D) Growth in soft agar of SK-LMS-1 cells expressing the indicated shRNAs. Foci were stained with MTT and counted. Data are presented as mean ± SD; n = 4. (TIF) [file pgen.1006752.s006.tif]

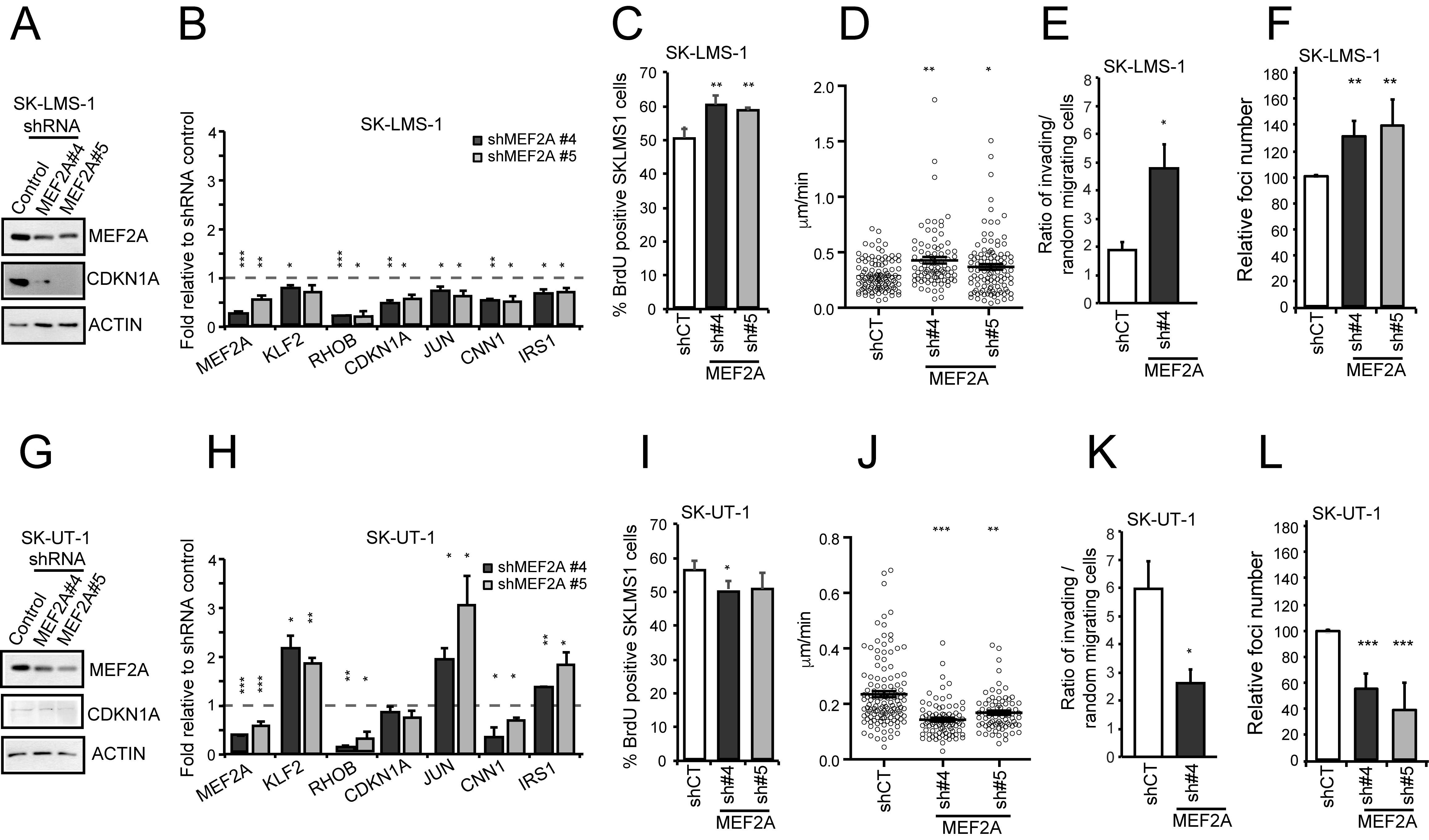

Supplement: S3 Fig — A) MEF2A expression was silenced by lentiviral infection using two different shRNA (#4 and #5). Immunoblot analysis of MEF2D and CDKN1A levels in SK-LMS-1 cells expressing the control shRNA or two different shRNAs against MEF2A. Actin was used as loading control. B) qRT-PCR analysis of the mRNA expression levels of MEF2A and of MEF2-target genes (KLF2, RHOB, CDKN1A, JUN, CNN1, IRS1) in SK-LMS-1 cells expressing the different shRNAs. mRNA levels are relative to control shRNA. Data are presented as mean ± SD; n = 4. C) Analysis of the cells synthetizing DNA as scored after BrdU staining. Data are presented as mean ± SD; n = 3. D) SK-LMS-1 cells expressing the indicated shRNAs were subjected to time-lapse analysis for 6 hours. Results represent the individual migration rate and the average (bar) from at least 140 cells from three independent experiments. Cell movements were quantified using MetaMorph software (Molecular Devices, Sunnyvale, CA). E) Invasion properties of the SK-LMS-1 cells expressing the shRNA4 against MEF2A or the control. Data are presented as mean ± SD; n = 4. F) Growth in soft agar of SK-LMS-1 cells expressing the indicated shRNAs, foci were stained with MTT and counted. Data are presented as mean ± SD; n = 4. G) MEF2A expression was silenced by lentiviral infection using two different shRNA (#4 and #5). Immunoblot analysis of MEF2D and CDKN1A levels in SK-UT-1 cells expressing the control shRNA or two different shRNAs against MEF2A. Actin was used as loading control. H) qRT-PCR analysis of the mRNA expression levels of MEF2A and of MEF2-target genes (KLF2, RHOB, CDKN1A, JUN, CNN1, IRS1) in SK-UT-1 cells expressing the different shRNAs. mRNA levels are relative to control shRNA. Data are presented as mean ± SD; n = 4. I) Analysis of the cells synthetizing DNA as scored after BrdU staining. Data are presented as mean ± SD; n = 3. J) SK-UT-1 cells expressing the indicated shRNAs were subjected to time-lapse analysis for 6 hours. Results represent the [file pgen.1006752.s007.tif]

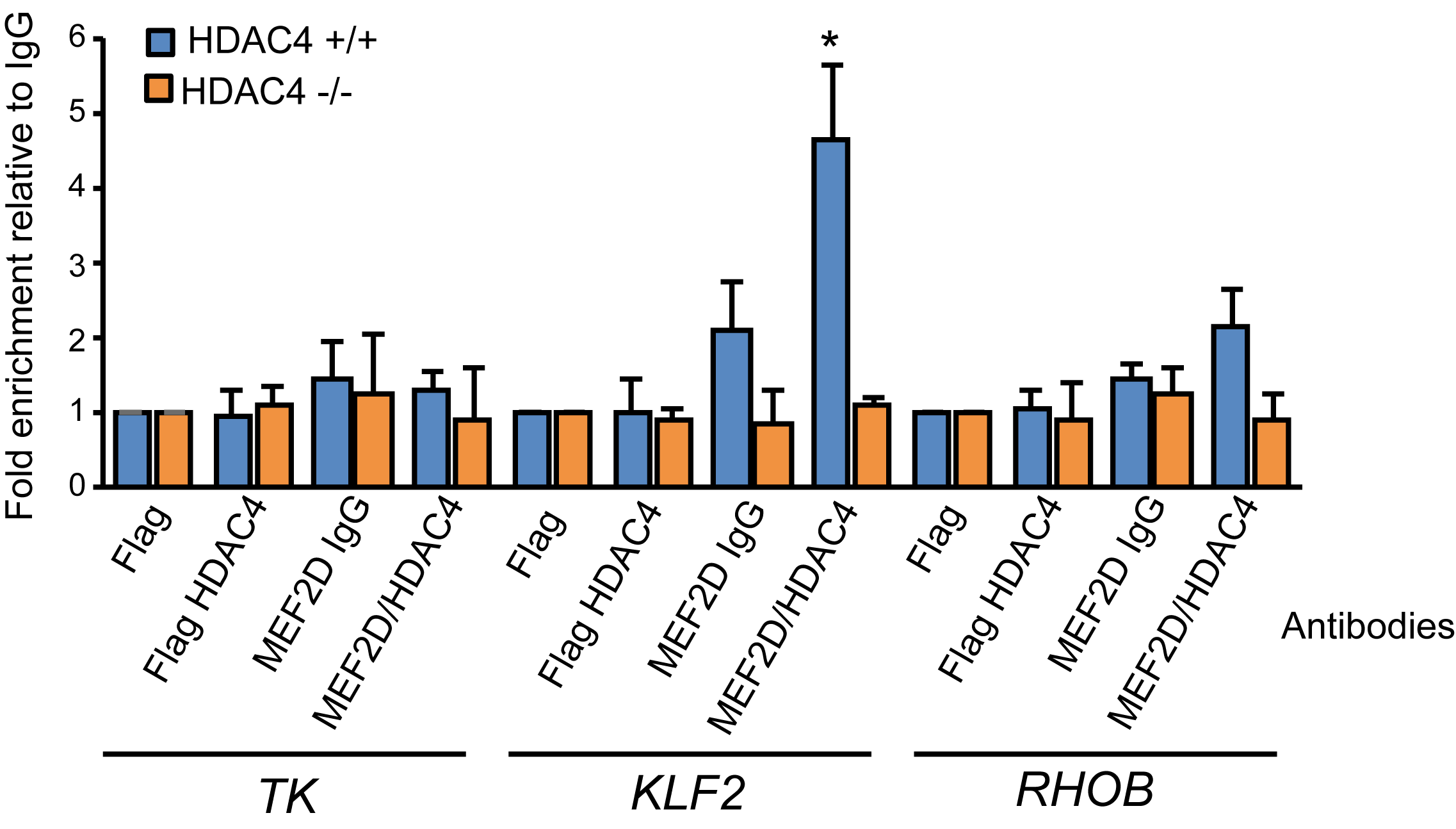

Supplement: S4 Fig — 6x10^6^ cells were employed. First immunoprecipitations were conducted ON with 2μg of anti-MEF2D or anti-FLAG antibodies. Protein-DNA complexes were collected with 8μl of protein A magnetic beads (ZymoMag, Zymo research) and washed twice with RIPA and TE. Beads were incubated for 30’ at 37°C in Re-Chip elution buffer (1×TE, 2%SDS, 15mM DTT), diluted 15 times into RIPA buffer and subjected to the second immunoprecipitation using 3μg of anti-HDAC4 or USP33 IgG as control. TK promoter was used as negative control. Data are presented as mean fold enrichment relatively to the first input (TIF) [file pgen.1006752.s008.tif]

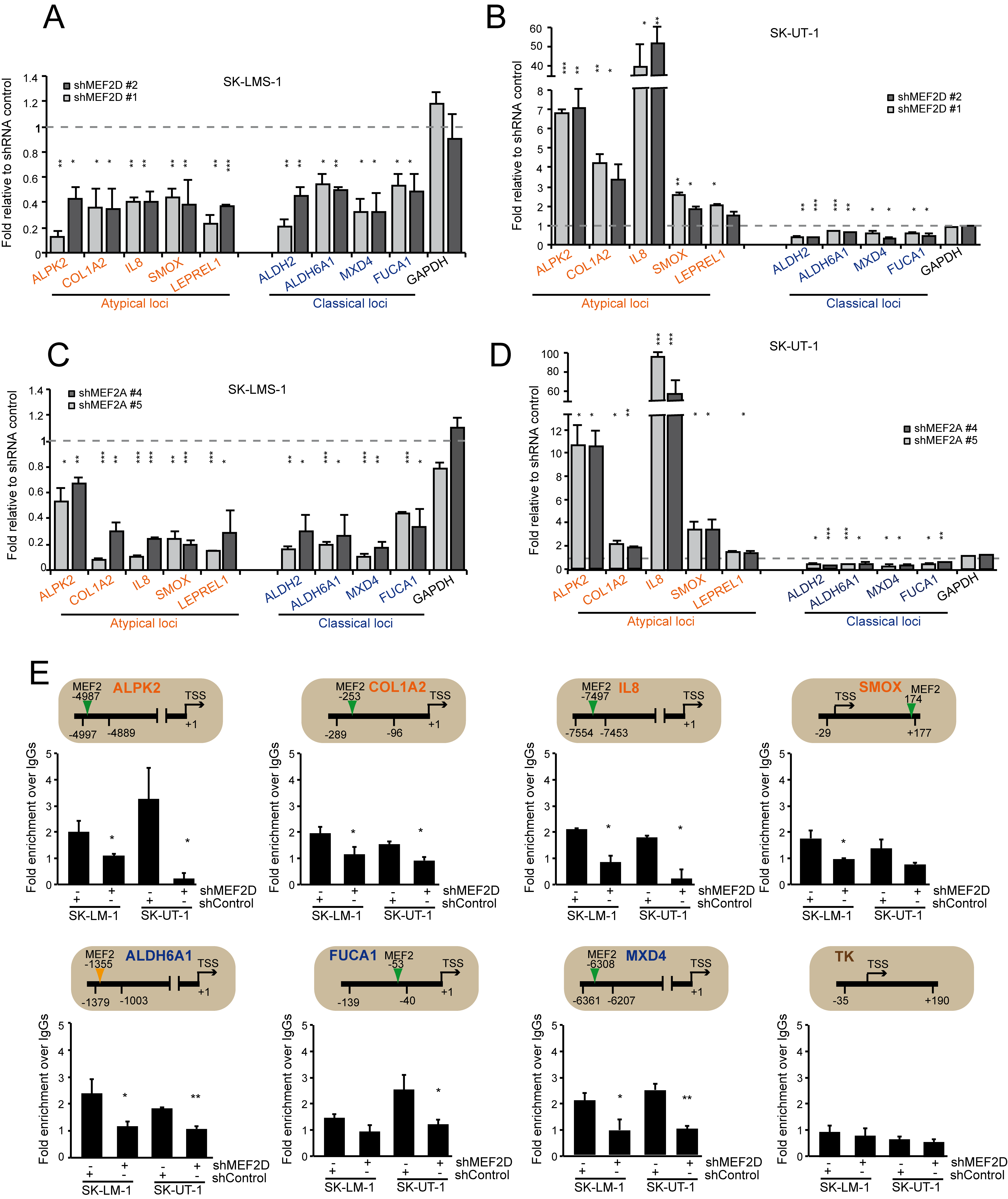

Supplement: S5 Fig — A) qRT-PCR analysis of the mRNA expression levels of the identified atypical and classical MEF2-target genes in SK-LMS-1 cells expressing the shRNAs against MEF2D. mRNA levels are relative to control shRNA. GAPDH was used as control. Data are presented as mean ± SD; n = 3. B) qRT-PCR analysis of the mRNA expression levels of the identified atypical and classical MEF2-target genes in SK-UT-1 cells expressing the shRNAs against MEF2D. mRNA levels are relative to control shRNA. GAPDH was used as control. Data are presented as mean ± SD; n = 3. C) qRT-PCR analysis of the mRNA expression levels of the identified atypical and classical MEF2-target genes in SK-LMS-1 cells expressing the shRNAs against MEF2A. mRNA levels are relative to control shRNA. GAPDH was used as control. Data are presented as mean ± SD; n = 3. D) qRT-PCR analysis of the mRNA expression levels of the identified atypical and classical MEF2-target genes in SK-UT-1 cells expressing the shRNAs against MEF2A. mRNA levels are relative to control shRNA. GAPDH was used as control. Data are presented as mean ± SD; n = 3. E) Chromatin was immunoprecipitated from SK-LMS-1 or SK-UT-1 cells using the anti-MEF2D antibody. Anti-FLAG antibody was used as control. Cells KD for MEF2D are indicated. TK promoter was used as negative control. The MEF2 binding site (arrowheads), the amplified region and the TSS (arrows) are indicated for each tested gene. Atypical MEF2-target genes are in orange whereas classical ones are in blue. Data are presented as mean ± SD; n = 3. (TIF) [file pgen.1006752.s009.tif]

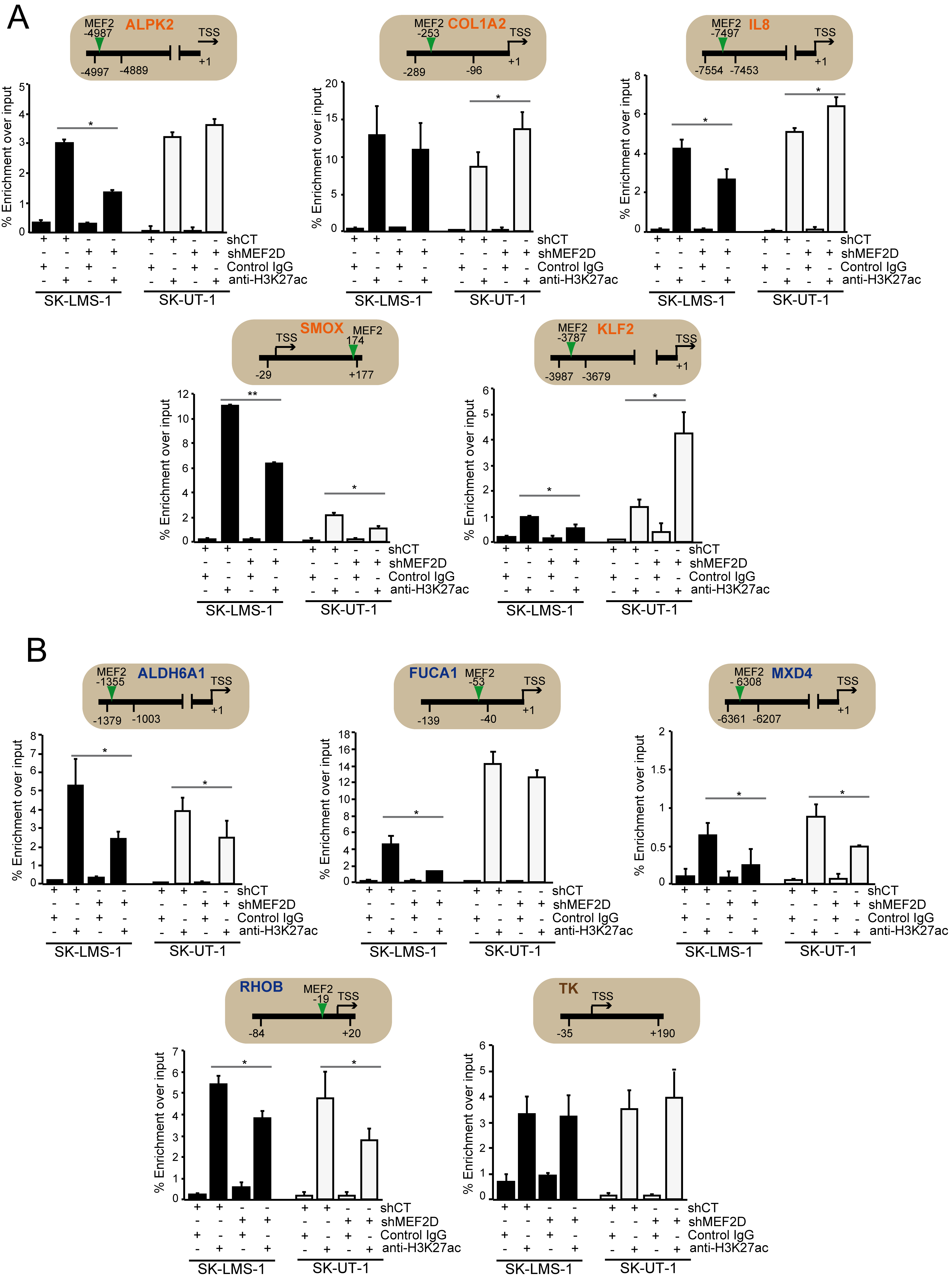

Supplement: S6 Fig — A) Chromatin was immunoprecipitated from SK-LMS-1 or SK-UT-1 cells WT or KD for MEF2D, using the anti-H3K27ac antibody. Normal rabbit IgGs were used as control. The MEF2 binding site (arrowheads), the amplified region and the TSS (arrows) are indicated for each tested atypical gene. Data are presented as mean ± SD; n = 3. B) Chromatin was immunoprecipitated from SK-LMS-1 or SK-UT-1 cells WT or KD for MEF2D, using the anti-H3K27ac antibody. Normal rabbit IgGs were used as control. TK promoter was used as negative control. The MEF2 binding site (arrowheads), the amplified region and the TSS (arrows) are indicated for each tested classical gene. Data are presented as mean ± SD; n = 3. Atypical MEF2-target genes are in orange whereas classical ones are in blue. (TIF) [file pgen.1006752.s010.tif]

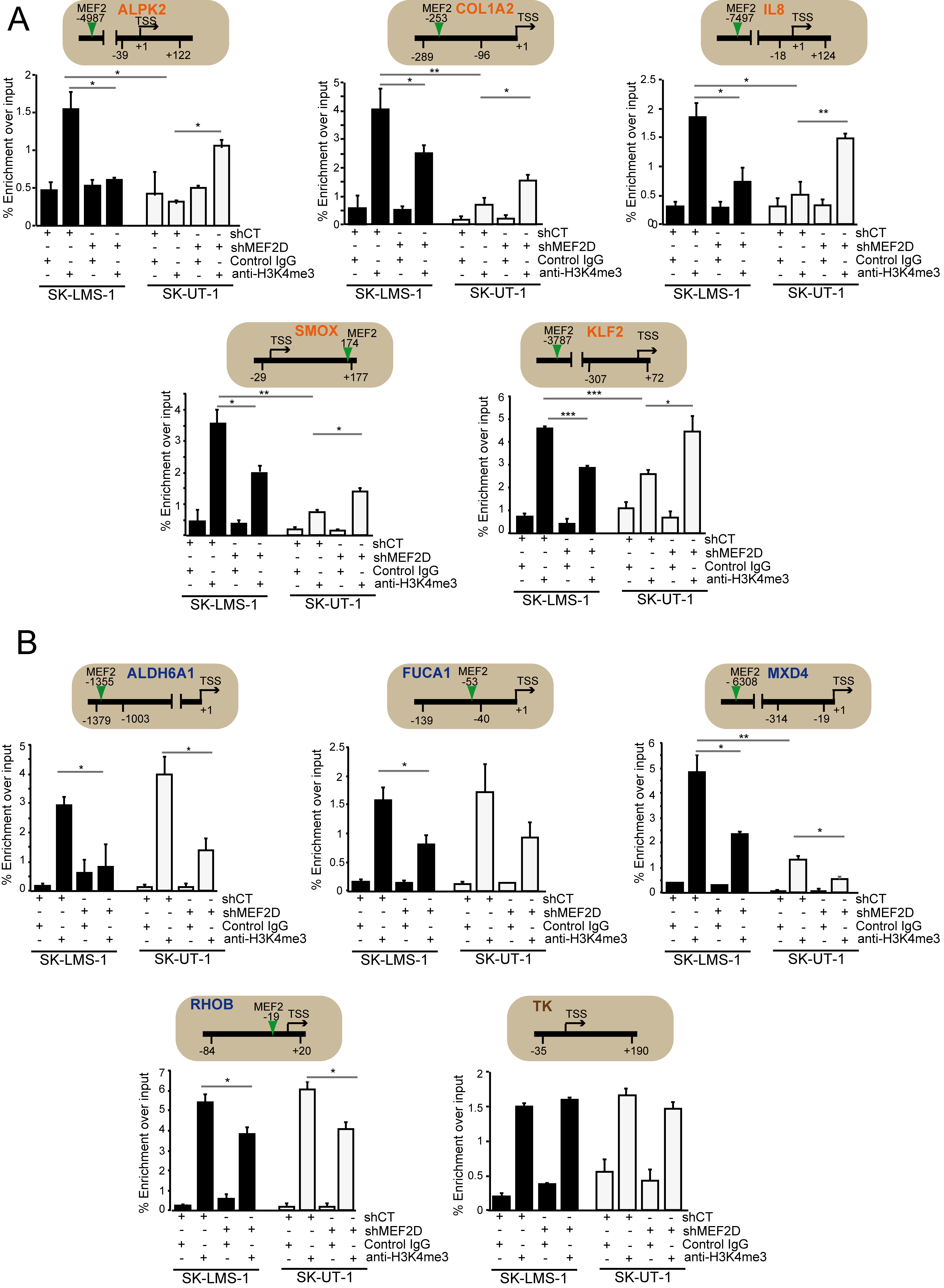

Supplement: S7 Fig — A) Chromatin was immunoprecipitated from SK-LMS-1 or SK-UT-1 cells WT or KD for MEF2D, using the anti-H3K4me3 antibody. Normal rabbit IgGs were used as control. The MEF2 binding site (arrowheads), the amplified region and the TSS (arrows) are indicated for each tested atypical gene. Data are presented as mean ± SD; n = 3. B) Chromatin was immunoprecipitated from SK-LMS-1 or SK-UT-1 cells WT or KD for MEF2D, using the anti-H3K4me3 antibody. Normal rabbit IgGs were used as control. TK promoter was used as negative control. The MEF2 binding site (arrowheads), the amplified region and the TSS (arrows) are indicated for each tested classical gene. Data are presented as mean ± SD; n = 3. Atypical MEF2-target genes are in orange whereas classical ones are in blue. (TIF) [file pgen.1006752.s011.tif]

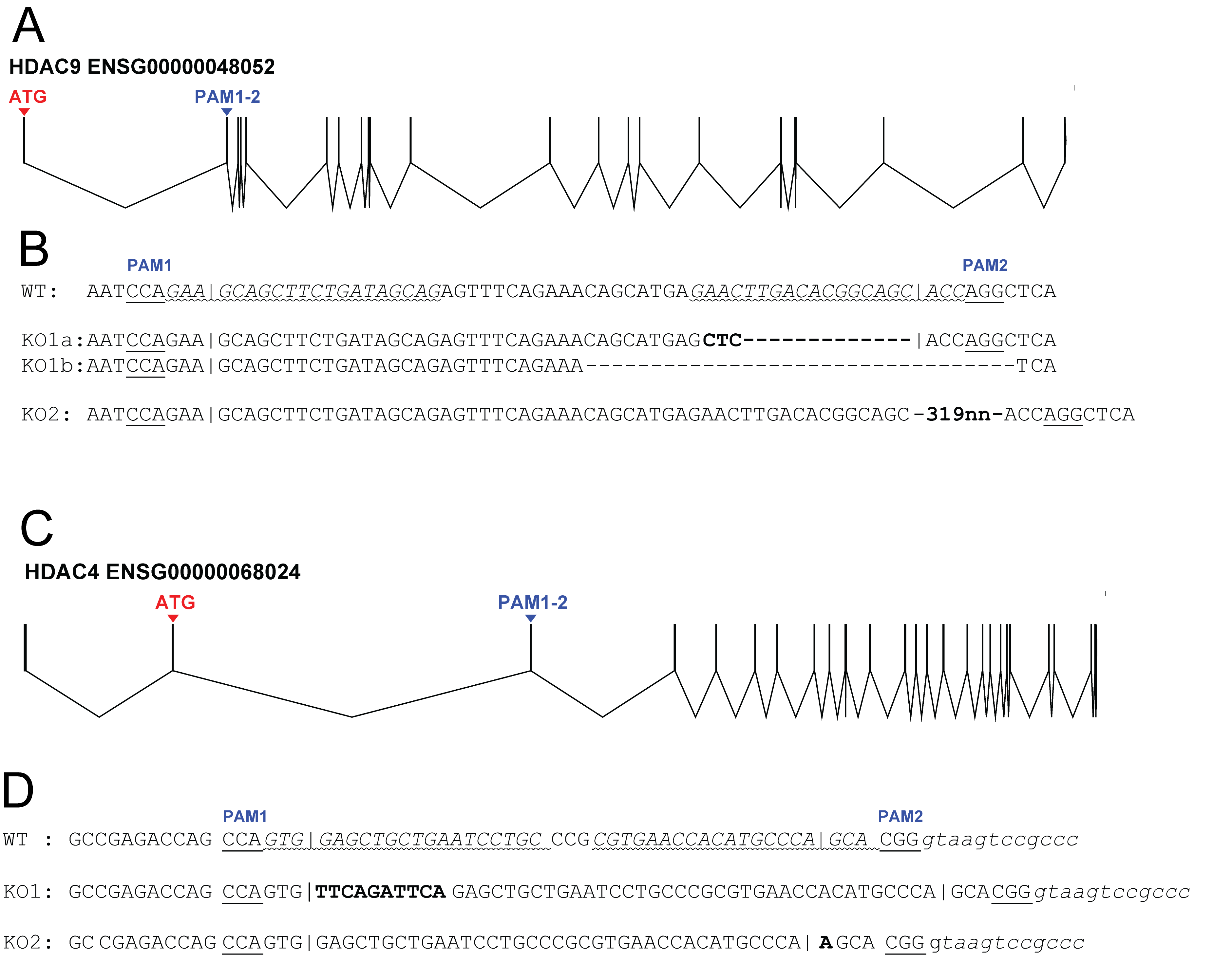

Supplement: S8 Fig — A) Schematic representation of HDAC9 genomic organization with indicated: the exons (vertical bars), the introns (junctions between the bars) and the PAM sequences utilized for the CRISPR approach. B) Genomic sequences of the HDAC9-/- SK-UT-1 cells used in this study. The sequence of HDAC9 genomic region targeted by the CRISPR/Cas9D10A is included. The PAMs and the two gRNAs are underlined. SKUT-1 HDAC9 KO clones were obtained through the delivery of the D10A mutant of SpCas9. Two sgRNAs designed on the second coding exon of HDAC9 were co-delivered to obtain two close cleavages on the genome to simulate a DSB (sgRNA1: CTGCTATCAGAAGCTGCTTC; sgRNA2: GAACTTGACACGGCAGCACC). Five clones were selected for the presence of deletions or insertion and among them the indicated two were selected for the analysis. C) Schematic representation of HDAC4 genomic organization with indicated: the exons (vertical bars), the introns (junctions between the bars) and the PAM sequences utilized for the CRISPR approach. D) Genomic sequences of the HDAC4-/- SK-UT-1 cells used in this study. The sequence of HDAC4 genomic region targeted by the CRISPR/Cas9 is included. The PAMs and the two gRNAs are underlined. SKUT-1 HDAC4 KO clones were obtained through the delivery of wild-type spCas9 (pLENTI-CRISPRv2). Two sgRNAs designed on the second coding exon were used (sgRNA1: GCAGGATTCAGCAGCTCCAC; sgRNA2: CGTGAACCACATGCCCAGCA). One and three KO clones were obtained respectively with sgRNA1 and 2. The two representative clones indicated here were selected for the analysis. (TIF) [file pgen.1006752.s012.tif]

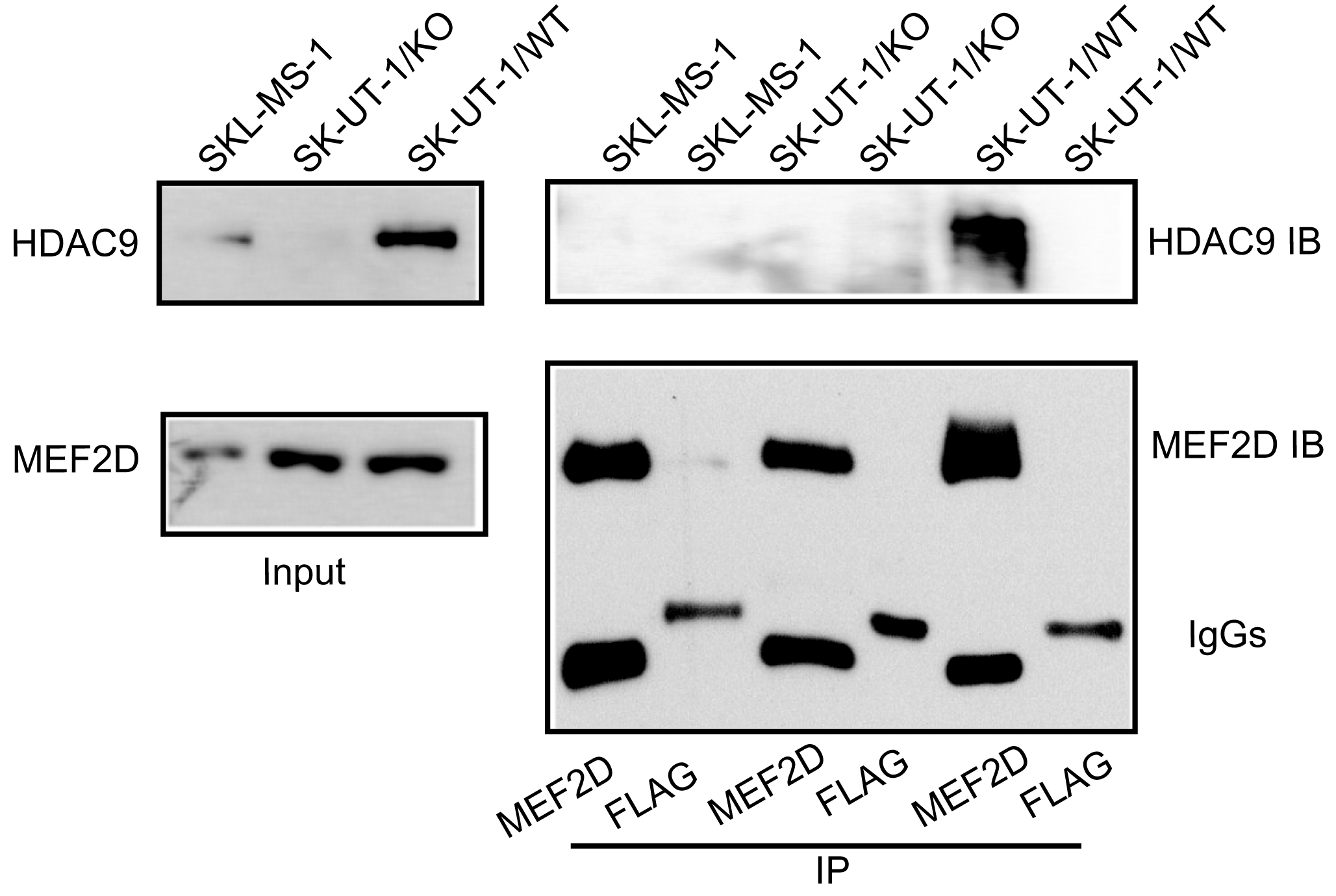

Supplement: S9 Fig — The MEF2D-HDAC9 complexes were immunoprecipitated from the different cell lines using 1μg of anti-MEF2D, or anti-FLAG antibodies, as a control. Immunocomplexes were subjected to immunoblotting using the anti-MEF2D and HDAC9 antibodies. (TIF) [file pgen.1006752.s013.tif]
